# The fire ant social chromosome supergene variant Sb shows low diversity but high divergence from SB

**DOI:** 10.1111/mec.14054

**Published:** 2017-04-01

**Authors:** Rodrigo Pracana, Anurag Priyam, Ilya Levantis, Richard A. Nichols, Yannick Wurm

**Affiliations:** ^1^ School of Biological and Chemical SciencesQueen Mary University of LondonMile End RoadLondonE1 4NSUK

**Keywords:** diversity in supergene variants, evolutionary strata, social chromosomes, *Solenopsis invicta*, queen number in ants

## Abstract

Variation in social behaviour is common, yet little is known about the genetic architectures underpinning its evolution. A rare exception is in the fire ant *Solenopsis invicta*: Alternative variants of a supergene region determine whether a colony will have exactly one or up to dozens of queens. The two variants of this region are carried by a pair of ‘social chromosomes’, SB and Sb, which resemble a pair of sex chromosomes. Recombination is suppressed between the two chromosomes in the supergene region. While the X‐like SB can recombine with itself in *SB/SB* queens, recombination is effectively absent in the Y‐like Sb because *Sb/Sb* queens die before reproducing. Here, we analyse whole‐genome sequences of eight haploid *SB* males and eight haploid *Sb* males. We find extensive SB–Sb differentiation throughout the >19‐Mb‐long supergene region. We find no evidence of ‘evolutionary strata’ with different levels of divergence comparable to those reported in several sex chromosomes. A high proportion of substitutions between the SB and Sb haplotypes are nonsynonymous, suggesting inefficacy of purifying selection in Sb sequences, similar to that for Y‐linked sequences in XY systems. Finally, we show that the Sb haplotype of the supergene region has 635‐fold less nucleotide diversity than the rest of the genome. We discuss how this reduction could be due to a recent selective sweep affecting Sb specifically or associated with a population bottleneck during the invasion of North America by the sampled population.

## Introduction

In a supergene, different allelic combinations at tightly linked loci determine different morphs in a population. Classical crossing studies initially led to the discovery of several such systems including supergenes controlling shell colour in the snail *Cepaea nemoralis* (Cain *et al*. 1960), wing pattern in *Papilio* butterflies (Clarke & Sheppard 1971) and heterostyly in the self‐incompatibility system of *Primula* (Mather 1950; Dowrick 1956). Recent advances in sequencing technology have allowed more rapid discovery and description of supergene regions, including those controlling the wing pattern mimicry of a butterfly species (Joron *et al*. 2011), alternate forms of social organization in ants (Wang *et al*. 2013; Purcell *et al*. 2014), reproductive morphs in birds (Küpper *et al*. 2015; Lamichhaney *et al*. 2015; Tuttle *et al*. 2016) and ecotypes in a flowering plant (Lowry & Willis 2010). Supergene evolution is thought to involve selection on alleles at two or more loci (Bull 1983). This selection acts to prevent the formation of disadvantageous combinations of alleles by suppressing recombination within the supergene region, for example by favouring the spread of inversions (Linksvayer *et al*. 2013; Schwander *et al*. 2014; Thompson & Jiggins 2014).

In this study, we focus on the evolution of a chromosome system responsible for two forms of social organization in the fire ant *Solenopsis invicta* (Wang *et al*. 2013). Colonies have either exactly one queen or up to dozens of reproductive queens, with multiple physiological, morphological and behavioural traits differing between the two social forms (Ross & Keller 1995; DeHeer *et al*. 1999; Keller & Ross 1999; Goodisman *et al*. 2000; DeHeer 2002; Buechel *et al*. 2014; Huang & Wang 2014). Queens that will form their own single‐queen colony typically disperse over greater distances and can effectively colonize newly available habitats. In contrast, multiple‐queen colonies can outcompete single‐queen colonies in saturated habitats and harsh environments and can split by budding (Herbers 1986; Nonacs 1993; Bourke & Heinze 1994; Ross & Keller 1995). These patterns of selection likely maintain both social forms within the species (Nonacs 1993; Ross & Keller 1995). The social dimorphism is genetically determined by a single Mendelian element (Keller & Ross 1998; Ross & Keller 1998; Krieger & Ross 2002; Ross & Keller 2002), recently shown to be a large (∼13 Mb) chromosome region (Wang *et al*. 2013). Recombination is suppressed between the two variants of this region which are carried by a pair of ‘social chromosomes’, SB and Sb. The region spans approximately 55% of the chromosomes and includes up to 600 protein‐coding genes (based on the genome sequence of an *SB* male; Wang *et al*. 2013). The two chromosomes differ by one large inversion affecting most of the region, and at least one further smaller inversion (48 kb) within the region. Recombination between SB and Sb is thought to have been lost relatively recently (less than 500 000 years ago; Wang *et al*. 2013). Indeed, SB and Sb contain largely the same protein‐coding gene content, although it is unclear how much allelic divergence there is between the two variants (Wang *et al*. 2013). The features of the region are consistent with it being a supergene. Although we use this term, we note two caveats: First, experimental evidence demonstrating that two or more loci contribute to differences between social forms is still lacking. Additionally, the mutations responsible for these differences may have occurred after the evolution of suppressed recombination in the region.

In single‐queen colonies, all workers and the queen have the *SB/SB* genotype. In contrast, multiple‐queen colonies include *SB/SB* and *SB/Sb* workers, but all reproductive queens are *SB/Sb* because workers kill *SB/SB* queens reaching reproductive maturity (Keller & Ross 1998; Ross & Keller 1998, 2002; Wang *et al*. 2013). Recombination occurs only in queens because fire ant workers are completely sterile and males are haploid (Tschinkel 2006). The recombination of the supergene region has two additional restrictions. First, the supergene region of SB is thought to recombine only in homozygous *SB/SB* queens of single‐queen colonies because the region does not recombine in heterozygote queens. A second restriction on recombination occurs because *Sb/Sb* queens die before reproducing (Ross 1997; DeHeer *et al*. 1999; Keller & Ross 1999; Gotzek & Ross 2007). If this genotype is always lethal, recombinants between two Sb haplotypes cannot be transmitted to the next generation (Wang *et al*. 2013). These restrictions on recombination are comparable to those affecting an X/Y sex chromosome system in a diploid species, with SB resembling an X chromosome, Sb resembling a nonrecombining Y chromosome, and the region outside the supergene resembling a pseudo‐autosomal region. Sb is the only part of the genome that is present exclusively in multiple‐queen colonies, whereas gene flow occurs extensively between colony types in the rest of the genome (albeit with a possible directional bias from single‐queen to multiple‐queen colonies; Ross 1992; Ross & Shoemaker 1993; Shoemaker & Ross 1996; Ross *et al*. 1997). The evolutionary effects of reduced recombination and lower effective population size compared to typical autosomes (Charlesworth & Charlesworth 2000) have been extensively studied in sex chromosomes, which can be seen as special cases of supergenes (Charlesworth 2016). These findings generate predictions for the fire ant system which can be tested by comparisons within and among SB and Sb genomes.

Fire ants have a haplo‐diploid sex determination system (Tschinkel 2006), and thus, it is possible to unambiguously distinguish SB and Sb haplotypes by sequencing haploid males. Here, we compare whole‐genome sequences of eight *SB* and eight *Sb* males to test predictions based on our understanding of supergene and sex chromosome evolution. First, we test whether there is sequence differentiation between the two chromosomes over the whole extent of the supergene region, indicating long‐term inhibition of recombination over the entire region. This would contrast with several large genomic inversions in *Drosophila melanogaster* (>7 Mb; Corbett‐Detig & Hartl 2012; Huang *et al*. 2014; Kapun *et al*. 2014) where recombination is suppressed in the regions near the breakpoints, but recombination in the form of gene conversion and double crossover events can occur in most of the inverted region (Navarro *et al*. 1997; Kapun *et al*. 2014). Second, we investigate whether the supergene region has lower genetic diversity than the rest of the genome, expecting a mild reduction in the X‐like SB due to its decreased effective population size (Betancourt *et al*. 2004; Hutter *et al*. 2007; Keinan *et al*. 2009; Vicoso & Charlesworth 2009; Hammer *et al*. 2010; Lambert *et al*. 2010; Arbiza *et al*. 2014), and a much stronger reduction in the Y‐like Sb due to strong Hill–Robertson effects in the absence of recombination (Kaiser & Charlesworth 2009; Wilson Sayres *et al*. 2014). These effects could also have led to degeneration of Sb – comparable to that observed in Y (or W) chromosomes, which can be detected by comparison of genomic sequence between chromosomes and among species (Charlesworth & Charlesworth 2000; Charlesworth *et al*. 2005; Bergero & Charlesworth 2009; Bachtrog 2013). The social chromosome supergene system may give insight into the early stages of degeneration of a nonrecombining region (Zhou *et al*. 2012) given the relatively young age of the system (Wang *et al*. 2013). Finally, we test whether the supergene region can be divided into strata with different levels of divergence between SB and Sb. In sex chromosome systems, strata are understood to represent discrete increases in the size of the sex‐linked region, possibly through the fixation of new structural mutations (Bergero & Charlesworth 2009). Strata have been documented in mammalian and avian sex chromosomes of relatively ancient origin (Lahn & Page 1999; Handley *et al*. 2004; Cortez *et al*. 2014; Wright *et al*. 2014; Zhou *et al*. 2014), and also in younger sex chromosomes in plants (Bergero *et al*. 2007; Wang *et al*. 2012; Papadopulos *et al*. 2015). The discovery of strata could be valuable in reconstructing the evolution of the social chromosome, particularly if an older ‘core’ region could be identified, as that would be expected to contain loci playing key roles in the determination of social form.

## Materials and methods

### Placing and orienting reference genome scaffolds using RADseq linkage maps

Our analysis uses the publicly available reference genome assembly of *S. invicta* (GCA_000188075.1; Si_gnG), produced by Wurm *et al*. (2011) based on the genome of a single *SB* male. Rather than providing a sequence for each of 16 chromosomes, this assembly includes 10 543 scaffolds (N50 size of 721 kb). We used allmaps (version 0.6.9; Tang *et al*. 2015b) to order and orient these scaffolds relative to seven equally weighted linkage maps that had previously been generated (Wang *et al*. 2013) using restriction site‐associated DNA sequencing (RADseq). Three of the maps were from different single‐queen families, and four were from different multiple‐queen families. To eliminate low‐confidence scaffolds, we removed scaffolds represented in only one single‐queen family linkage map if the scaffold contained fewer than four markers in this map. We identified 43 scaffolds with markers in multiple linkage groups. These scaffolds are likely artefactual chimeras of noncontiguous sequences but could also include structural differences between the individual used for genome assembly and the families used for linkage mapping. We split each of these scaffolds, retaining only the portions within the ranges of the markers mapping to each of the linkage groups. In total, we mapped 249.8 Mb (63.1%) of the reference genome assembly to linkage groups. We identified the RADseq markers that cosegregate with the *Gp‐9* locus (a diagnostic marker of the supergene region; Keller & Ross 1998; Ross & Keller 1998, 2002) in the four linkage maps of multiple‐queen families. As in Wang *et al*. (2013), all of the RADseq markers that cosegregate with the *Gp‐9* marker were located in scaffolds placed in linkage group 16.

### Samples, sequencing and sequence filtering

Our sequences are from eight different multiple‐queen colonies that were initially collected in the field in Texas and Georgia, USA, and subsequently modified in the laboratory to ensure that each colony contains only a single *SB/Sb* queen. One *SB* haploid male and one *Sb* haploid male (i.e. full brothers) were taken from each of these colonies. In 2010, high‐coverage sequence was first produced from an *SB* male (NCBI SAMN00014755, ∼35× coverage; Wurm *et al*. 2011) and subsequently from an *Sb* male (NCBI SRX206834, ∼69× coverage; Wang *et al*. 2013). The remaining males were sequenced at 6×‐8× coverage in a single batch in 2012 (NCBI SRP017317; Wang *et al*. 2013. seqtk (version 1.0r31; https://github.com/lh3/seqtk) was used to trim 5 bp from the left and right ends of the reads of the high‐coverage *SB* sample, 3 bp from the left and right ends of the reads of the high‐coverage *Sb* sample and 10 bp from the left and the right ends of the reads of all low‐coverage samples. The use of three different trimming criteria was necessary because of the different qualities of reads obtained in the three different sequencing runs. Additionally, our trimming was more stringent with the low‐coverage samples because the influence of sequencing errors can be higher with low‐coverage data. We removed all reads where fewer than 75% of the bases had a quality score larger than 20 using fastq_quality_filter (version 0.0.13.2; http://hannonlab.cshl.edu/fastx_toolkit/). To reduce the size and complexity of the data set, we subsampled the data from the high‐coverage individuals to retain only ∼25× genome coverage from each. This data reduction strategy was applied to increase processing speed and reduce computational complexity (a higher number of reads with sequencing errors increase the complexity of a de Bruijn graph; Brown *et al*. 2012). The subsampling threshold was subjectively chosen after inspection of alignments indicated that the older, higher coverage data had lower read qualities and higher coverage heterogeneity than the newer lower‐coverage data. Mapping the reads to the reference genome with bowtie2 (version 2.1.0; Langmead & Salzberg 2012) showed that the majority of the genome is covered: by combining the data from all samples, >79% of the reference genome had a mean coverage ⩾100×. This level of coverage is sufficient to reliably identify variable sites across the genome. Furthermore, the data set provides sufficient predictive power to genotype the vast majority of sites in most individuals because >91% of the genome had ⩾1× coverage in each of the 16 samples, with ⩾94% of coding sequence covered at ⩾1× coverage in each of the 16 samples. Our use of haploid individuals for this study allows us to accurately call genotypes even with low coverage and removes the need to infer haplotype of origin (Cortez *et al*. 2014).

### Variant calling with the Cortex reference‐free genotyper

To identify variants and call genotypes for the 16 individuals, we used the de Bruijn graph‐based genotyper cortex (version 1.0.5.20; Iqbal *et al*. 2012) with the bubble caller algorithm (run_calls.pl) and options: k‐mers 31 and 61, reference‐free variant identification and genotyping, ‘auto_cleaning’, ‘apply_pop_classifier’ and ‘dups’. Cortex identified 939 006 variants with a PASS tag. We filtered these in line with recommendations from the Cortex authors. Specifically, we retained sites with site confidence SITE_CONF >15, genotype confidence GT_CONF >10 for all low‐coverage individuals and GT_CONF >2 for the high‐coverage individuals. Filtering by SITE_CONF retains sites nonambiguously classified as true variants and not as errors or repeats, while filtering by GT_CONF retains sites with nonambiguous genotyping for all individuals. These filters preferentially retained variant calls with high‐coverage support (Fig. S1, supporting information). Finally, we removed 335 sites where the coverage for the called allele was 0 in one or more individuals after filtering. 628 476 variant sites remained after all filters were applied. For the analysis of diversity within each genotype group (the group of *SB* and the group of *Sb* samples), we applied the GT_CONF filter and the coverage filter separately for each group. This was because we did not want the quality of the genotype calls for individuals in one group to affect the diversity estimates of the individuals in the other group. The number of single nucleotide polymorphisms (SNPs) and insertions and deletions (indels) in each data set after filtering is shown in Table S1 (supporting information).

### Variant calling in short tandem repeat (STR) loci

We identified short tandem repeat (STR) loci in the reference assembly using tandem repeats finder (version 4.07b; Benson 1999) with options: Minscore=20 and MaxPeriod=6. We limited the analysis to STRs with a reference repeat unit number larger than the following thresholds: 9 for mononucleotide STRs, 5 for dinucleotide STRs and 4 for larger STRs. We used lobstr (version 3.0.3; Gymrek *et al*. 2012) with default parameters to identify variant STR loci among the 14 low‐coverage individuals. We kept only those variant calls where all individuals had quality value Q⩾0.25, at least two reads supporting the called allele and a maximum coverage of 25 reads. We applied these filters separately for each genotype group in the analyses of diversity within each group. The number of polymorphic STR sites in each of the data sets is given in Table S2 (supporting information).

### Population genetics measurements

Using a custom r script (version 3.0.2; R Core Team 2013) with bioconductor packages (Gentleman *et al*. 2004) including genomicranges, variantannotation and genomicfeatures, we divided the genome into overlapping sliding windows and estimated different measures of divergence and diversity. To define a sliding window of a given size, we skipped nonassembled positions (represented by N in the reference genome sequence). We analysed 30 kb sliding windows every 10 kb, 150 kb windows every 50 kb, 10 kb windows every 5 kb and 10 kb windows every 10 kb. We counted the number of SNPs with fixed differences between the group of eight males with the *SB* genotype and the group of eight males with *Sb* genotypes and used hierfstat (Goudet 2005) to measure multilocus $F_{\mathrm{ST}}$ between the two groups (Yang 1998). Reported numbers refer only to scaffolds mapped to linkage groups (we identify five unmapped scaffolds that are also likely part of the supergene but these were not considered for population‐genetic analyses). Our samples consisted of pairs of brothers, with each pair including one *SB* and one *Sb* individual. Each individual was thus most closely related to its brother in the other group. Consequently the estimates of between‐group differentiation were often smaller than within‐group differentiation, leading to slightly negative $F_{\mathrm{ST}}$ values (outside the supergene region). The estimates of differentiation could be sensitive to the sampling of individuals for each group. We evaluated this effect with permutation tests, which randomly reallocated individuals in the analysis. These permutation tests show that there is differentiation in the supergene region when each group includes subsets of individuals of alternate genotypes, but not when individuals of different genotypes are randomly assigned to the same group (Fig. S9, supporting information). We also measured nucleotide diversity *π* (Nei 1987) within each group in each window, using all sites (coding and noncoding). Within each of the two groups, we compared *π* in the supergene region with *π* in the remaining mapped genome.

### Manual inspection of sites variable among *Sb* individuals

Only 54 sites in the supergene region (10.8 Mb) were variable among the eight *Sb* individuals. Assuming that the false‐positive error rate of the variant calling pipeline is constant along the genome, we would expect that false‐positive variants account for a disproportionately high number of the variants called in regions with low diversity. We therefore investigated whether any of these 54 variant sites were false positives. For this, we produced whole‐genome alignments for each *SB* and *Sb* individual using bowtie2 (version 2.1.0; Langmead & Salzberg 2012). We manually inspected the alignments at each of these sites using igv (version 2.3.47; Robinson *et al*. 2011) and concluded that at least 41 of the sites are truly variable among *Sb* individuals. The remaining 13 sites were false‐positive polymorphisms (Table S3, supporting information). We manually inspected 50 other polymorphic sites randomly chosen from our complete data set, and we found no similar errors.

### Annotation of protein‐coding genes

We downloaded *Solenopsis invicta* protein‐coding gene annotation release 100 from NCBI (14 464 protein‐coding genes, taking the longest isoform for each gene). To eliminate potential pseudogenes from our analysis, we removed 2101 genes that had the tags ‘partial=true’ or ‘the sequence of the model RefSeq transcript was modified relative to this genomic sequence to represent the inferred CDS’; we similarly removed 22 genes for which the coding sequence either included an N? character in the reference genome assembly or for which the length was not a multiple of three; we removed 13 mitochondrial genes that had the tag ‘transl_table=5’. We removed genes with insertions or deletions in any of the samples (159 genes, six of which mapped to the supergene region). Of the 12 169 genes that remained after filtering, 424 were located in the supergene and 8975 in the remaining mapped region.

### Synonymous and nonsynonymous divergence (*dS* and *dN*)

For each protein‐coding gene in the supergene region, we created one representative consensus sequence for SB and one for Sb based on fixed differences between the two groups of individuals. Using seqinr (version 3.0‐7; Charif & Lobry 2007), we estimated the synonymous and nonsynonymous divergence per site (*dS* and *dN*, respectively) between the consensus gene sequences of the two supergene variants.

### Evolutionary strata in the supergene region

To determine whether the divergence between SB and Sb was greater in some parts of the supergene region than in others, we examined (i) $F_{\mathrm{ST}}$, (ii) the density of SNPs with fixed differences (i.e. SNP positions where SB and Sb were fixed for alternative alleles), (iii) differences in the number of repeat units at positions where there were short tandem repeats (STRs) at which SB and Sb were fixed for alternative alleles and (iv) *dS*. We used Welch *t*‐tests to measure the difference in *dS* between any group of at least 30 neighbouring genes and all other genes (Fig. S13B, supporting information). The group of protein‐coding genes with the lowest *dS* relative to the remaining genes was designated as a putative young stratum. We then tested whether this difference in *dS* was significant. For this, we performed 10 000 simulations using the observed substitution rate of one substitution per 3000 bp of coding sequence (see Fig. S13A, supporting information); for each gene, we generated a random number of substitutions using a Poisson distribution with the parameter value equal to the gene length multiplied by the substitution rate (Nei & Kumar 2000). For each simulated data set, we repeated the analysis using the Welch *t*‐test to identify the group of genes showing the greatest differentiation. We then recorded the distribution of the uncorrected *P*‐values from the corresponding *t*‐tests, for comparison with the value obtained with the observed data.

### Evidence for reduced efficacy of purifying selection

We first tested whether the ratio of nonsynonymous to synonymous divergence (*dN/dS*) in the protein‐coding genes of the supergene region is different from a genome‐wide background distribution of *dN/dS* values. We obtained this distribution by comparing two species of leafcutter ant, *Atta cephalotes* and *Acromyrmex echinatior*, which shared a common ancestor less than 15 million years ago and are in the same subfamily (Myrmicinae) as *S. invicta* (Moreau & Bell 2013). For this, we downloaded coding sequences from *A. cephalotes* geneset 1.2 (Suen *et al*. 2011) and *A. echinatior* geneset 3.8 (Nygaard *et al*. 2011) from Fourmidable (Wurm *et al*. 2009). orthodb 5 (Waterhouse *et al*. 2013) identified 9690 one‐to‐one orthologs between the two species. After prank codon‐level alignment of each pair of orthologs (version 120626; Löytynoja & Goldman 2008), we obtained a *dN/dS* value for each pair using paml codeml (version 4.5; Yang 2007). We used a Wilcoxon signed rank test to test whether the *S.  invicta dN/dS* values measured for the genes located in the supergene region differ from the *dN/dS* distribution measured from the comparison between *A. cephalotes* and *A. echinatior*. We additionally tested whether the proportion of nonsynonymous to synonymous substitutions between SB and Sb in the genes located the supergene region is equal to the proportion of nonsynonymous to synonymous polymorphisms among the SB individuals in the same genes (McDonald & Kreitman 1991).

### Gene ontology analysis

We functionally annotated the filtered protein‐coding gene annotations using interproscan (version 5.15‐54.0; Jones *et al*. 2014), which produced Gene Ontology (GO) annotations for each gene (Ashburner *et al*. 2000). We divided the genes in the supergene region into two sets, one composed of genes with one or more fixed different nonsynonymous SNPs between the *SB* and the *Sb* individuals, the other with no such differences. We tested whether any GO term is enriched in the set of genes with nonsynonymous differences relative to the other set (using goatools version 0.5.9; Tang *et al*. 2015a). After Bonferroni correction, there was no significantly enriched GO term.

## Results

### Strong genetic differentiation between SB and Sb

**Figure 1:**
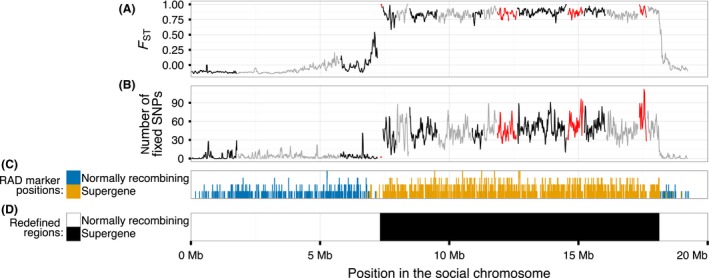
Genetic differentiation between eight *SB* and eight *Sb* individuals along the social chromosome. (A) $F_{\mathrm{ST}}$ and (B) number SNPs with a fixed difference between the two groups in 30 kb sliding windows with a 10 kb step; alternating colours represent different scaffolds; the orientation of the scaffolds in red is unknown. (C) Position of RADseq SNPs in each scaffold that mark the social chromosome in linkage map studies of four multiple‐queen families (Wang *et al*. 2013). (D) The extent of the redefined supergene region.

We analysed whole‐genome sequences of eight *SB* and eight *Sb* haploid males originating from the invasive North American population of *S. invicta* (Wurm *et al*. 2011; Wang *et al*. 2013). To test whether SB and Sb are differentiated in the supergene region, we measured multilocus $F_{\mathrm{ST}}$ (Yang 1998) between the two groups of males using a sliding window approach. We discovered that linkage group 16 includes a large region (10.8 Mb) where all windows have high $F_{\mathrm{ST}}$ ($0.6⩽F_{\mathrm{ST}}⩽1$), flanked by windows with sharply reduced $F_{\mathrm{ST}}$ (Fig. 1A for 30 kb windows with a 10 kb step; Fig. S2–S4, supporting information, for other window sizes). The region with high $F_{\mathrm{ST}}$ largely overlaps with the supergene region identified by lower‐resolution linkage maps of families from multiple‐queen colonies (Wang *et al*. 2013)(Fig.  1C). In the mapped genome outside the supergene region, 99.5% of windows had $F_{\mathrm{ST}}\;<\;0.25$ between the two groups of males, and no other genomic segment included consecutive nonoverlapping windows with $F_{\mathrm{ST}}\;>\;0.25$ (Fig. S5, supporting information).

Differentiation between SB and Sb can also be measured in terms of fixed differences, the positions where all *SB* individuals carry one allele and all *Sb* individuals carry another. As expected, almost all SNP sites with fixed differences between the two groups (15 367 of 15 404, or 99.8%) were located in the region with high $F_{\mathrm{ST}}$ (Fig. 1B, Tables 1 and  S1, supporting information), a highly significant enrichment given that this region made up only 6% of the mapped assembly ($\chi^{2}$ test, $\chi_{d.f.\;=\;1}^{2}=$ 338  815, $P\;<\;10^{-15}$; see Fig. S2–S4, supporting information, for other window sizes and Fig. S6, supporting information, for comparison to other linkage groups). All 111 STR sites in mapped scaffolds with fixed differences between the two groups were also located in the region (Table S2, supporting information), despite it containing only 4% of the polymorphic STR sites in the mapped assembly. Permutation tests showed that the high number of sites with fixed differences between the two groups was not an artefact of grouping the individuals (Fig. S9, supporting information).

**Table 1:** Summary of the differentiation between the *SB* and *Sb* groups and the diversity within each group in the supergene region (only in mapped scaffolds) and the in rest of the mapped genome. $F_{\mathrm{ST}}$ and *π* are given as the means of values across nonoverlapping 10 kb windows

	Supergene region	Remaining mapped genome
Size		
Size in assembly	10.8 Mb	239 Mb
Differentiation		
$F_{\mathrm{ST}}$ ± SD	0.84 ± 0.11	−0.06 ± 0.07
SNPs with fixed differences between the *SB* and the *Sb* males	15 367 (1.4 per 1000 bp)	37 (0.0002 per 1000 bp)
Number of SNPs		
Among *SB*	16 033 (1.5 per 1000 bp)	442 014 (1.8 per 1000 bp)
Among *Sb*	34 (0.0031 per 1000 bp)	433 862 (1.8 per 1000 bp)
Nucleotide diversity		
Among *SB*	Mean $\pi \;=\;7\;\times \;10^{-4}$	Mean $\pi \;=\;8\;\times \;10^{-4}$
Among *Sb*	Mean $\pi \;=\;1\;\times \;10^{-6}$	Mean $\pi \;=\;8\;\times \;10^{-4}$

These results suggest that the differentiation between SB and Sb affects the entirety of the supergene region. This result contrasts with the pattern found around inversions in other species, including several examples in *Drosophila melanogaster*, where differentiation is limited to the region around the inversion breakpoints (Navarro *et al*. 1997; Kapun *et al*. 2014). The divergence between the two groups in terms of fixed differences at synonymous sites of protein‐coding sequence (*dS*) was low (mean of 0.002 ± standard deviation of 0.0028; Table 2), consistent with a recent origin of the supergene system. Based on the reference genome assembly of an *SB* male used in this analysis, the redefined supergene region is 10.8 Mb long and forms 56% of the assembly mapped to linkage group 16 (Fig. 1D, Table 1). We consider the rest of the linkage group to be normally recombining, similar to the pseudo‐autosomal region of sex chromosome pairs.

**Table 2:** Synonymous and nonsynonymous SB–Sb divergence (based on SNPs with fixed differences between the *SB* and the *Sb* males), compared to the number of polymorphisms of each type among the *SB* males. *dN* and *dS* are, respectively, the number of synonymous and nonsynonymous substitutions per synonymous and nonsynonymous site. *pN* and *pS* are, respectively, the number of synonymous and nonsynonymous polymorphisms per synonymous and nonsynonymous site

	Nonsynonymous SNP	Synonymous SNP	Proportion
Fixed different sites			
In the supergene (SB–Sb)	374 (*dN* = 0.0008)	417 (*dS* = 0.002)	*dN*/*dS* = 0.38
Outside the supergene	0 (*dN* = 0)	1 (*dS* = 0)	*dN*/*dS* = 0
Polymorphisms among *SB*			
In the supergene (SB)	209 (*pN* = 0.0005)	490 (*pS* = 0.0022)	*pN*/*pS* = 0.24
Outside the supergene	4815 (*pN* = 0.0005)	11614 (*pS* = 0.0022)	*pN*/*pS* = 0.23

### Assigning five further scaffolds to the supergene region

Our analysis included only the portion of the reference assembly mapped to linkage groups (250 Mb of 396 Mb). To investigate whether any of the 188 large (>100 kb) unmapped scaffolds in the assembly could also be part of the supergene region, we tested whether any of these scaffolds have windows with high $F_{\mathrm{ST}}$ (>0.75) and a high density of SNPs with fixed differences between the two groups of males (>25 sites per 30 kb window; Fig. S10, supporting information). We found five such large unmapped scaffolds; they included 56.3 % (996 of 1770) of the SNP sites with fixed differences between groups among unmapped scaffolds. Permutation tests again showed that the high number of fixed differences between the two groups is not an artefact of grouping the individuals (Fig. S11, supporting information). Including the five unmapped scaffolds increases the supergene region to 11.8 Mb. This is smaller than previously determined from four linkage maps (12.7 Mb; Wang *et al*. 2013), possibly due to the inability of RAD linkage maps to accurately separate markers in the supergene region from cosegregating markers in linkage disequilibrium. The reference assembly (396 Mb) is missing portions of the genome that are difficult to assemble: the genome size is estimated to be between 463 and 753 Mb (Li & Heinz 2000; Johnston *et al*. 2004; Ardila‐Garcia *et al*. 2010; Wurm *et al*. 2011). Assuming an even distribution of the nonassembled genome and the unmapped scaffolds not analysed in our study, the size of the supergene region could be between 19.4 and 31.5 Mb.

### No evidence for strata in the social chromosome system

**Figure 2:**
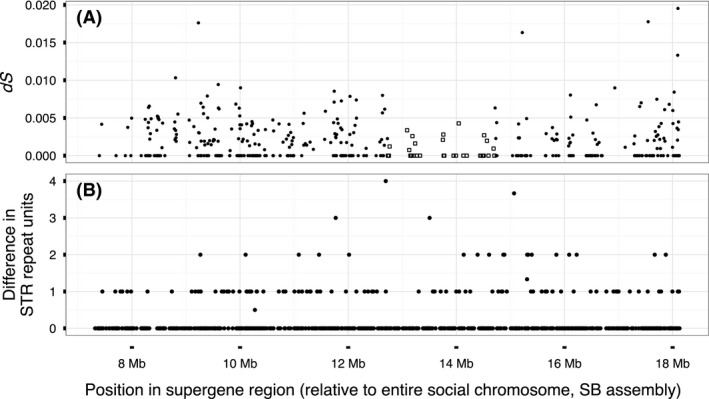
No evidence for evolutionary strata in the supergene region. (A) The rate of synonymous substitution (*dS*) of protein‐coding genes in the region; the squares show the group of genes with lower *dS* than the rest. (B) Difference in the number of repeat units between *SB* and *Sb* individuals in STR sites; figure includes nonpolymorphic STR sites and sites with a fixed difference between the two groups.

Many sex chromosomes can be divided into strata with distinct levels of divergence between the X and the Y, each reflecting a discrete event of loss of recombination (Lahn & Page 1999; Handley *et al*. 2004; Bergero *et al*. 2007; Wang *et al*. 2012; Cortez *et al*. 2014; Zhou *et al*. 2014). We studied the variation in divergence along the supergene region, investigating whether any segment of the region has a different level of divergence relative to the rest of the region. We used four measures of divergence: $F_{\mathrm{ST}}$ (Fig. 1A), the number of SNPs with fixed differences between the two groups in 30 kb windows (Fig. 1B), the synonymous substitution rate (*dS*; Fig. 2A) and the difference in the number of STR units between the two groups in STRs with alternative alleles fixed in SB and in Sb (Fig. 2B). Of these, only *dS* showed a segment with different divergence than the rest of the supergene. This segment included 36 genes with lower *dS* (0.0007 ± 0.0012) than the other genes in the region (0.0021 ± 0.0029; Fig. 2B). This pattern might be taken to indicate that the region with the lowest *dS* forms a younger stratum than the rest of the supergene. Using simulations, we tested whether this pattern could be explained by a null model of uniform random differentiation across the supergene region. In 1164 of 10 000 (12%) simulations, the test statistic (the *P*‐value from a two‐sided Welch t‐test comparing the cluster of least differentiated loci with the remainder) was stronger than the one we observed ($P\;=\;4\times 10^{-7}$), and thus we conclude that the observed difference in *dS* is not significant (Materials and Methods; Fig. S13, supporting information). In line with this conclusion, the density of SNPs with fixed differences between the two groups of males was actually higher, not lower, in the region overlapping the genes with low *dS* (1.8 SNPs with fixed differences per kbp) than in the rest of the supergene (0.82 SNPs with fixed differences per kbp). Furthermore, the number of STR sites with fixed differences in the region with low *dS* (14 of 177 STR sites, 8%) was similar to the rest of the supergene (97 of 856, 11%; Fisher's exact test, *P* = 0.23; Fig. 2B).

As an additional test, we divided the supergene region into 10 kb windows which we ranked by the number of SNPs with fixed differences between the group of *SB* males and the group of *Sb* males. This analysis identified no strata with nonoverlapping ranges of these counts (Fig. S12B, supporting information). Ranking the genes in the supergene region by their rate of synonymous substitution (*dS*) between SB and Sb produced a similar result (Fig. S12C, supporting information).

In summary, we found no convincing evidence for strata of differentiation. This suggests that a single event led to suppression of recombination over the whole region. Alternatively, the divergence between SB and Sb or the amount of time between potential successive events leading to recombination suppression in the region may be insufficient to allow the detection of strata (Chibalina & Filatov 2011; Papadopulos *et al*. 2015).

### Low genetic diversity among *Sb* individuals

**Figure 3:**
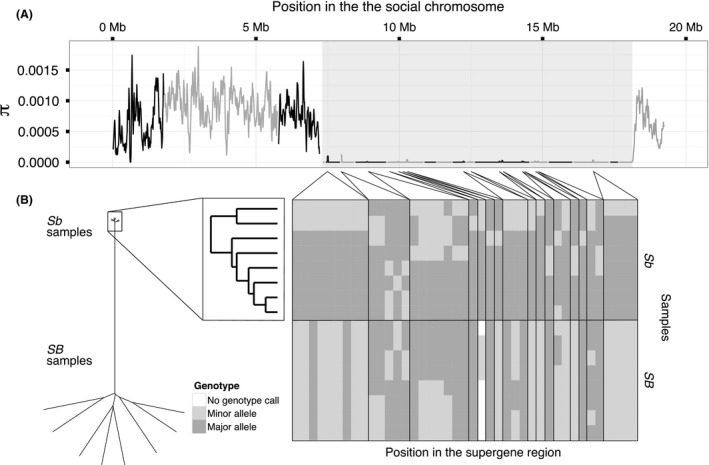
Diversity among *Sb* individuals in the supergene region of the social chromosome. (A) Nucleotide diversity (*π*) among *Sb* individuals using 30 kb sliding windows with a 10 kb step. Alternating colours represent different scaffolds; the orientation of the scaffold with the hashed line is unknown; the background shading indicates the extent of the supergene region. (B) Genotypes of positions variable among the *Sb* individuals in the supergene region: dendrogram of the Euclidean distances between the genotypes of all individuals (left) and of the *Sb* individuals only (right); heat map showing the genotypes at each position (classed as major and minor relative to the *Sb* samples only).

Among *Sb* individuals, nucleotide diversity in the supergene region only 0.16% (i.e. a 635‐fold reduction) of its level in the rest of the genome (mean $\pi \;=\;1.3\times 10^{-6}$ vs. $\pi \;=\;8.2\times 10^{-4}$ among the *Sb* individuals in nonoverlapping 10 kb windows; one‐sided Wilcoxon rank‐sum test, *W* = 220 732, $P\;<\;10^{-16}$; Fig. 3A, Table 1; see Fig. S2–S4, supporting information, for other window sizes and Fig. S7, supporting information, for comparison to other linkage groups). Indeed, only 41 sites in the supergene region were variable among *Sb* individuals (34 SNPs and 7 indels; 0.0038 variants per kbp; Table 1), 0.18% of the density of the rest of the genome (2.2 variants per kbp; $\chi^{2}$ test, $\chi_{d.f.\;=\;1}^{2}=$ 23 293, $P\;<\;10^{-16}$; Tables 1 and  S1, supporting information). Similarly, none of the 1779 STR sites present in the region were polymorphic among *Sb* individuals, while 12 118 of 44 107 STR sites in the rest of the mapped genome were polymorphic (Table S2, supporting information). Hierarchical clustering based on Euclidean distances between *Sb* sequences formed two groups (Fig. 3B), but these are not distinct haplotypes. Nine of the variant sites had alleles that were shared among individuals in both groups, while twelve were also polymorphic among the *SB* individuals.

**Figure 4:**
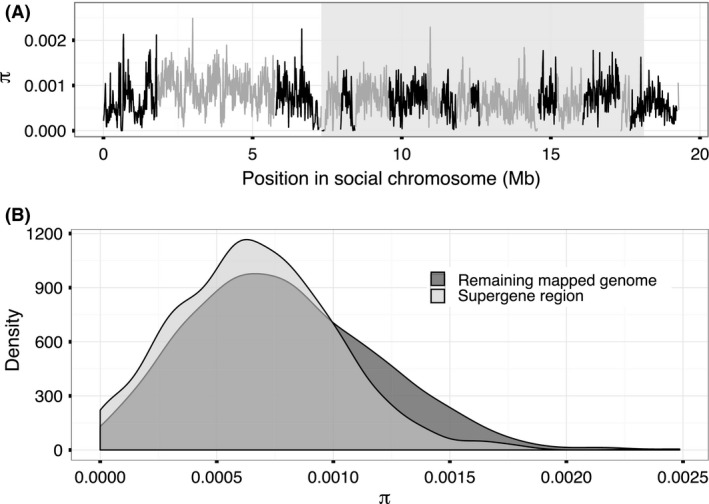
Nucleotide diversity (*π*) among *SB* individuals. (A) Distribution of *π* values along the social chromosome (30 kb windows with 10 kb step). Shading indicates the position of the supergene region. (B) Frequency density of *π* as measured in nonoverlapping 10 kb windows of the whole mapped genome.

In contrast to the *Sb* individuals, nucleotide diversity among *SB* individuals in the supergene region was 80% of the diversity of the rest of the genome (mean $\pi \;=\;6.7\times 10^{-4}$ vs. $\pi \;=\;8.4\times 10^{-4}$ among the *SB* individuals in nonoverlapping 10 kb windows; one‐sided Wilcoxon rank‐sum test, *W* = 8 488 848, $P\;<\;10^{-16}$; Fig. 4, Table 1; see Fig. S2–S4, supporting information, for other window sizes and Fig. S8, supporting information, for comparison to other linkage groups). There were 18 875 sites variable among *SB* individuals in the region (16 033 SNPs and 2842 indels; 1.7 variants per kbp), 79% of the density of the rest of the genome (2.2 variants per kbp; $\chi^{2}$ test, $\chi_{d.f.\;=\;1}^{2}=$ 988.6, $P\;<\;10^{-16}$; Tables 1 and  S1, supporting information ). The proportion of polymorphic STR sites in the region (537 of 2094 total STR sites in the region) was 91% of the proportion in the rest of the genome (12 950 of 45 803; $\chi^{2}$ test, $\chi_{d.f.\;=\;1}^{2}\;=\;988.6$, *P* = 0.01; Table S2, supporting information).

Although low genetic diversity in the Sb supergene variant may be expected because of Hill–Robertson effects, the magnitude of the reduction is striking (illustrated by the relatively shorter terminal branches at the top of the dendrogram in Fig. 3B). We outline possible reasons for the reduction of diversity in both supergene variants in the Discussion section.

### Reduced efficacy of purifying selection on protein‐coding sequences in Sb

Hill–Robertson interference is expected to reduce the efficacy of purifying selection in the nonrecombining Sb supergene variant (Bachtrog 2013; Wang *et al*. 2013). This effect could cause a reduction in the ability of selection to remove nonsynonymous mutations (which are mostly deleterious) from Sb. We performed two analyses to determine whether there is an enrichment of nonsynonymous substitutions in Sb. First, we determined that the ratio of nonsynonymous to synonymous divergence (*dN*/*dS*) for the genes in the supergene region (*dN*/*dS* = 0.38; Table 2) was significantly greater than a genome‐wide distribution of *dN*/*dS* calculated from 9664 one‐to‐one orthologs between the leafcutter ants *Atta cephalotes* and *Acromyrmex echinatior* (median *dN*/*dS* = 0.13; Wilcoxon signed rank test, *V* = 5 541 543, $P\;<\;2\times 10^{-16}$). The relative enrichment of nonsynonymous substitutions between SB and Sb in the supergene region suggests that at least one of the supergene variants has been affected by reduced efficacy of purifying selection. Second, we performed a McDonald–Kreitman test (McDonald & Kreitman 1991), which showed that the proportion of nonsynonymous substitutions between *SB* and *Sb* haplotypes was significantly greater than the proportion of nonsynonymous polymorphisms among *SB* individuals in the supergene region ($\chi^{2}$ test, $\chi_{d.f.\;=\;1}^{2}$ = 46.3, $P\;<\;10^{-11}$; Table 2). This result provides additional support for the hypothesis that the efficacy of purifying selection is reduced in the Sb variant of the supergene.

## Discussion

### Relative reduction of genetic diversity in the supergene variants

In a large population with equal numbers of males and females, there are three copies of the *X* and one copy of the *Y* for every four copies of each autosome (i.e. *X*/*A* = 3/4 = 75% and *Y*/*A* = 1/4 = 25%). In the fire ant supergene system, it is more challenging to estimate the proportion of each chromosome in the population. The relative contribution of multiple‐queen colonies and single‐queen colonies to future generations is not precisely known, but we can consider two extremes. On the one hand, multiple‐queen colonies have been found at ∼2× higher densities than single‐queen colonies (Glancey *et al*. 1987; Porter 1991; Macom & Porter 1996) and each multiple‐queen colony can include dozens of reproductive queens (Goodisman & Ross 1997). The number of *SB/SB* queens could thus be negligible in comparison with the number of *SB/Sb* queens. In this case, there would be similar numbers of SB and of Sb chromosomes. Excluding the effects of selection and linkage, the effective population size $N_{e}$ of the SB supergene variant would therefore be half that of the rest of the genome (*SB/other* = ∼50%). However, several arguments upport the other extreme, where the $N_{e}$ of the SB supergene variant is much larger than this. First, *SB/SB* queens are much better at dispersing and colonizing newly disturbed habitats than *SB/Sb* queens (Ross & Keller 1995). Disturbance in fire ant habitats is common (e.g., due to flooding; Ross & Keller 1995; Tschinkel 2006), which likely leads to a greater number of single‐queen colonies than multiple‐ queen colonies and in turn increases the number of SB relative to Sb. Perhaps more importantly, there is evidence of a bias in gene flow from single‐queen to multiple‐queen colonies (Ross 1992; Ross 1993; Ross & Shoemaker 1993; Shoemaker & Ross 1996; Ross *et al*. 1997; Shoemaker *et al*. 2006; Lawson *et al*. 2012). In the scenario where all new *SB/Sb* queens are the product of an *SB* male from a single‐queen colony fertilizing an Sb‐bearing egg, the effective population size of the SB supergene variant would reflect that of the local *SB/SB* populations from which it arrived – where it would be equal to the population size of other chromosomes (*SB/other* = ∼100%). Our analysis shows that the relative diversity of the SB supergene variant relative to other regions of the genome is substantially larger than 50% ($\pi_{SB}/\pi_{other}\;=\;80\%$). This suggests that the evolutionary history of the social chromosomes lies between the two extremes. A more detailed interpretation of this pattern will require quantification of the key parameters of gene flow, population size and mutation rate.

The Sb supergene variant can be compared to other nonrecombining regions, where Hill–Robertson interference strongly reduces genetic diversity. Even in the absence of selective sweeps, the effect of background selection has been modelled to reduce the effective population size of nonrecombining regions to ∼1.5% of normally recombining chromosomes under a range of reasonable parameter values (Kaiser & Charlesworth 2009). This prediction fits the values seen in the neo‐Y chromosome of *Drosophila miranda* (Bartolome & Charlesworth 2006), the fourth (dot) chromosome of *Drosophila melanogaster* (Wang *et al*. 2002; Sheldahl *et al*. 2003; Shapiro *et al*. 2007) and the human Y chromosome (Wilson Sayres *et al*. 2014). The particularly low diversity of the Sb supergene variant relative to the rest of the genome ($\pi_{Sb}/\pi_{other}\;=\;0.16\%$) implies that the effective population size of this supergene variant is much lower than the expectation that Sb/other = ∼1.5%. This discrepancy suggests that the Sb supergene variant went through a recent fixation event. It is unlikely that this fixation event was associated with the loss of recombination at the origin of the supergene, given the estimate that SB and Sb have been diverging for 350 000 to 424 000 years (based on a simple molecular clock model of *dS*; Wang *et al*. 2013). Mutations occurring since the split would have increased the genetic diversity among Sb variants of the supergene. Instead, the low diversity within the Sb variant could have been caused by a more recent fixation. This fixation could have happened by chance in a recent population bottleneck, or it could have occurred as a result of a recent selective sweep through a larger population or a recent introgression from another species.

The first of these explanations could apply to our samples, as they come from invasive North American fire ant populations. Migration from the native range in South America in the 1930s is inferred to have been associated with a strong population bottleneck (Shoemaker *et al*. 2006; Ross & Shoemaker 2008; Ascunce *et al*. 2011) with perhaps only 15–30 unrelated mated queens involved in the colonization of the entire introduced range (Ross & Shoemaker 2008). The origin of Sb in North America is unknown, although it is likely to have happened at a later date, with the existence of multiple‐queen colonies only explicitly documented since the 1970s (Fletcher *et al*. 1980; Shoemaker *et al*. 2006). Regardless of Sb origin, a bottleneck during the invasion of North America would have had a stronger effect in Sb relative to SB or the rest of the genome, as Sb is present in at most one copy per queen and completely absent from single‐queen colonies (Krieger & Ross 2005; Shoemaker *et al*. 2006). This bottleneck could potentially have been intensified by the relative inability of *SB/Sb* queens to found new colonies independently due to their lower body weight and fertility than *SB/SB* queens (Ross & Keller 1995). Indeed, multiple‐queen colonies tend to disperse by budding (Ross & Keller 1995), which may slow down the increase in population size of Sb after any bottleneck. Therefore, it is possible that the recent fixation of the Sb supergene variant happened during the North American invasion. Nevertheless, the fixation may instead have occurred in the source population before the invasion (Ascunce *et al*. 2011), either due to a population bottleneck or a selective sweep. It is also possible that the fixation of the Sb supergene variant results from introgression of the chromosome from another *Solenopsis* species (Keller 1995; Krieger & Ross 2005; Huang & Wang 2014), similarly to the introgression of wing pattern mimicry alleles in butterflies (Smith & Kronforst 2013). Determining the timing of the fixation event would require studying the diversity of the Sb supergene variant in larger numbers of samples of different *Solenopsis* species in the native South American range (Huang & Wang 2014): a selective sweep or introgression event acting on South American populations would decrease genetic diversity in those populations as well as in North America, while a bottleneck during migration would have decreased genetic diversity only in North America. This work would probably discover Sb haplotypes that we did not sample in our study, including those carrying the alleles that were previously seen in the *Gp‐9* locus (a diagnostic marker of the supergene region) in multiple‐queen colonies (Krieger & Ross 2005). Studying other *Solenopsis* species would allows us to infer the ancestral state of the supergene region, and furthermore inform us of whether all socially dimorphic species share the same supergene system and whether it has introgressed between species.

Assuming that the Sb supergene variant never recombines, all polymorphic sites affecting it would have originated via independent mutations since the putative fixation event, creating separate Sb lineages in the population. This pattern is not seen in our study. Instead, alleles are shared by different groups of individuals at each site, with some sites variable both in Sb and SB (Fig. 3B). It is possible that some of these polymorphic sites are located in incorrectly assembled portions of the genome, such as contigs incorrectly placed in scaffolds mapping to the supergene, or in mobile genetic elements that may be on different chromosomes in different individuals. Alternatively, the Sb polymorphisms may have been produced by parallel mutations or transferred between haplotypes by noncanonical recombination or gene conversion between the SB and Sb supergene variants (Navarro *et al*. 1997).

### Reduced efficacy of purifying selection in Sb

In an autosome, recombination allows each locus to respond independently to selection. In a nonrecombining chromosome such as a Y, selection on any locus affects the whole chromosome. This process causes a strong reduction in the efficacy of purifying selection, an effect that is thought to contribute to the degeneration of Y chromosomes (Charlesworth & Charlesworth 2000; Bachtrog 2013). The relative enrichment of nonsynonymous substitutions between SB and Sb suggests that Sb is affected by reduced efficacy of purifying selection. We lack the power to detect the extent to which positive selection on loci in SB or Sb contributed to this bias (Bachtrog 2004). However, we found no enrichment for particular gene functions (Gene Ontology terms) in the set of genes with nonsynonymous substitutions, which is consistent with the idea that most differences are not adaptive to a few specific functions. Despite the increased *dN*/*dS*, SB and Sb are more similar than X and Y in many sex chromosome systems that have been studied (Bachtrog 2013), for instance not being affected by widespread gene loss (Wang *et al*. 2013). This difference may be explained by the relatively recent divergence between SB and Sb, in the same way that the absence of gene loss in the neo‐Y of *Drosophila albomicans* is attributed to the very young age of that system (Zhou *et al*. 2012). However, it is also possible that purifying selection acting on haploid Sb males has some effect in slowing down Sb degeneration (which would be comparable to constraints reported in the haploid UV system of brown algae; Ahmed *et al*. 2014).

## Conclusion

We have characterized the recombination regime of the young supergene system of the fire ant, and associated effects on divergence and polymorphism. The results suggest straightforward studies that would resolve questions about the system's evolutionary origins. Future analysis of South American samples should clarify whether the recent fixation of Sb is the result of a bottleneck, a selective sweep, or a recent introgression from a different species. Another fruitful line of inquiry would be to obtain samples from adjacent single and multiple‐queen populations, in order to assess the relative importance of demography and selection on the genetic diversity in the SB region and the rest of the genome, and to quantify the size and direction of gene flow between the two social forms.

Y.W., R.P. and R.A.N. designed the study; R.P. performed most of the research under guidance from Y.W. and R.A.N; A.P. and I.L., respectively, helped with gene annotations and with gene set enrichment analysis. All authors wrote the manuscript. All authors gave final approval for publication.

## Data accessibility

DNA sequences: GenBank accessions SAMN00014755, SRX206834, SRP017317; Genotypes: Filtered VCF files are available at http://dx.doi.org/10.5061/dryad.js509; SB–Sb divergence in genes in the supergene region: fasta file with consensus Sb sequences and table with *dS* and *dN* are available at http://dx.doi.org/10.5061/dryad.js509; dS values for *Atta cephalotes* and *Acromyrmex echinatior* are available at http://dx.doi.org/10.5061/dryad.js509.

## Supporting information


**Fig. S1** Filtering the data by quality values and assembly quality removes sites with very low coverage calls.
**Fig. S2** Differentiation and diversity in linkage group 1 and linkage group 16 (the social chromosome) in 30 kb sliding windows with a step of 10 kb.
**Fig. S3** Differentiation and diversity in linkage group 1 and linkage group 16 (the social chromosome) in 15 kb sliding windows with a step of 50 kb.
**Fig. S4** Differentiation and diversity in linkage group 1 and linkage group 16 (the social chromosome) in 10 kb sliding windows with a step of 5 kb.
**Fig. S5** $F_{\mathrm{ST}}$ in 30 kb sliding windows with a step of 10 kb along scaffolds mapped to all linkage groups (LG1 to LG16).
**Fig. S6** Number of SNPs with a fixed difference per 30 kb sliding windows with a step of 10 kb along scaffolds mapped to all linkage groups (LG1 to LG16).
**Fig. S7** Nucleotide diversity among *Sb* individuals (*π*) in 30 kb sliding windows with a step of 10 kb along scaffolds mapped to all linkage groups (LG1 to LG16).
**Fig. S8** Nucleotide diversity among *SB* individuals (*π*) in 30 kb sliding windows with a step of 10 kb along scaffolds mapped to all linkage groups (LG1 to LG16).
**Fig. S9** We used permutation tests to ensure that the fixed differences between the group of *SB* individuals and the group of *Sb* individuals were not the result of an arbitrary grouping of individuals.
**Fig. S10** Signature of genetic differentiation between *SB* and *Sb* individuals in different regions of the genome.
**Fig. S11** Permutation tests showing that, in the scaffolds that putatively belong to the supergene region, we observe a high number of fixed differences only when we group the individuals by genotype.
**Fig. S12** Absence of strata with non‐overlapping ranges of divergence between *SB* and *Sb*.
**Fig. S13** Differences in the rate of synonymous substitutions (*dS*) between putative evolutionary strata in the real data and in an example of a simulation.
**Table S1** Number of variant sites detected with cortex (Iqbal et al. 2012) in each group of samples; number of variants with different alleles fixed in each of the *SB* and *Sb* groups.
**Table S2** Number of polymorphic and non‐polymorphic short tandem repeat (STR) sites detected with lobstr (Gymrek et al. 2012) in each group of samples; number of variants with different alleles fixed in each of the *SB* and *Sb* groups.
**Table S3** Visual inspection of SNP and indel positions variable among Sb individuals in the supergene region.
