# Supplementary material for: The fire ant social chromosome supergene variant Sb shows low diversity but high divergence from SB
Source: Mol Ecol. 2017 Apr 1;26(11):2864–79. doi: 10.1111/mec.14054 (PMC5485014; doi:10.1111/mec.14054)
Supplement: Supplementary file 1 — Fig. S1 Filtering the data by quality values and assembly quality removes sites with very low coverage calls. Fig. S2 Differentiation and diversity in linkage group 1 and linkage group 16 (the social chromosome) in 30 kb sliding windows with a step of 10 kb. Fig. S3 Differentiation and diversity in linkage group 1 and linkage group 16 (the social chromosome) in 15 kb sliding windows with a step of 50 kb. Fig. S4 Differentiation and diversity in linkage group 1 and linkage group 16 (the social chromosome) in 10 kb sliding windows with a step of 5 kb. Fig. S5 FST in 30 kb sliding windows with a step of 10 kb along scaffolds mapped to all linkage groups (LG1 to LG16). Fig. S6 Number of SNPs with a fixed difference per 30 kb sliding windows with a step of 10 kb along scaffolds mapped to all linkage groups (LG1 to LG16). Fig. S7 Nucleotide diversity among Sb individuals (π) in 30 kb sliding windows with a step of 10 kb along scaffolds mapped to all linkage groups (LG1 to LG16). Fig. S8 Nucleotide diversity among SB individuals (π) in 30 kb sliding windows with a step of 10 kb along scaffolds mapped to all linkage groups (LG1 to LG16). Fig. S9 We used permutation tests to ensure that the fixed differences between the group of SB individuals and the group of Sb individuals were not the result of an arbitrary grouping of individuals. Fig. S10 Signature of genetic differentiation between SB and Sb individuals in different regions of the genome. Fig. S11 Permutation tests showing that, in the scaffolds that putatively belong to the supergene region, we observe a high number of fixed differences only when we group the individuals by genotype. Fig. S12 Absence of strata with non‐overlapping ranges of divergence between SB and Sb. Fig. S13 Differences in the rate of synonymous substitutions (dS) between putative evolutionary strata in the real data and in an example of a simulation. Table S1 Number of variant sites detected with cortex (Iqbal et al. 2012) in each group of sample [file MEC-26-2864-s001.pdf]

The fire ant social chromosome supergene variant Sb shows low diversity but high divergence from SB

Supporting Information

Rodrigo Pracana[1], Anurag Priyam, Ilya Levantis, Richard A. Nichols and Yannick Wurm[2]
School of Biological and Chemical Sciences, Queen Mary University of London, United Kingdom
Corresponding authors: E-mail: [1]r.pracana@qmul.ac.uk, [2]y.wurm@qmul.ac.uk.

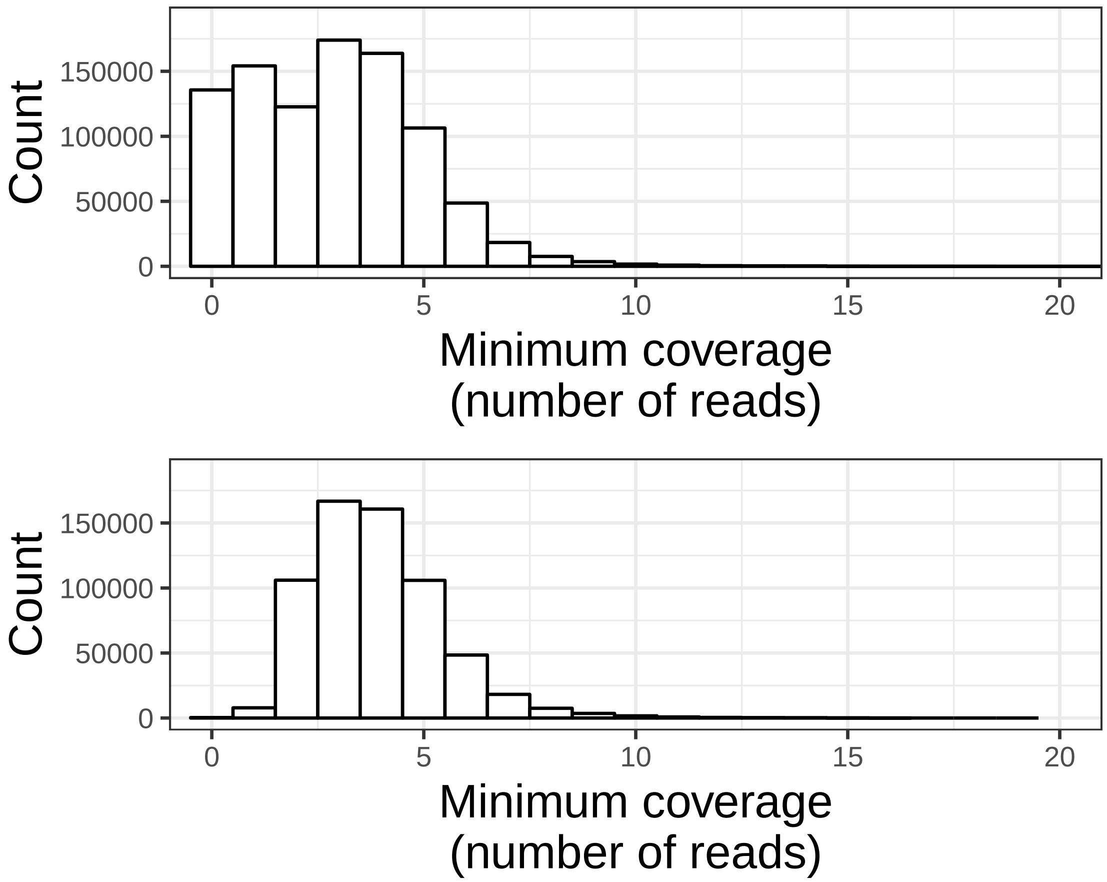

Figure S1: Filtering the data by quality values and assembly quality removes sites with very low coverage calls. The plot shows the distribution of the coverage of the individual with minimum coverage for each variant site before (top) and after (bottom) filtering by site confidence, genotype confidence and assembly quality. These filters had the effect of removing the majority (97.2%) of variant calls where the individual with the minimum coverage had coverage < 2. Of all the variant sites removed by these filters, 90.8% had an individual for which read coverage was smaller than 2.

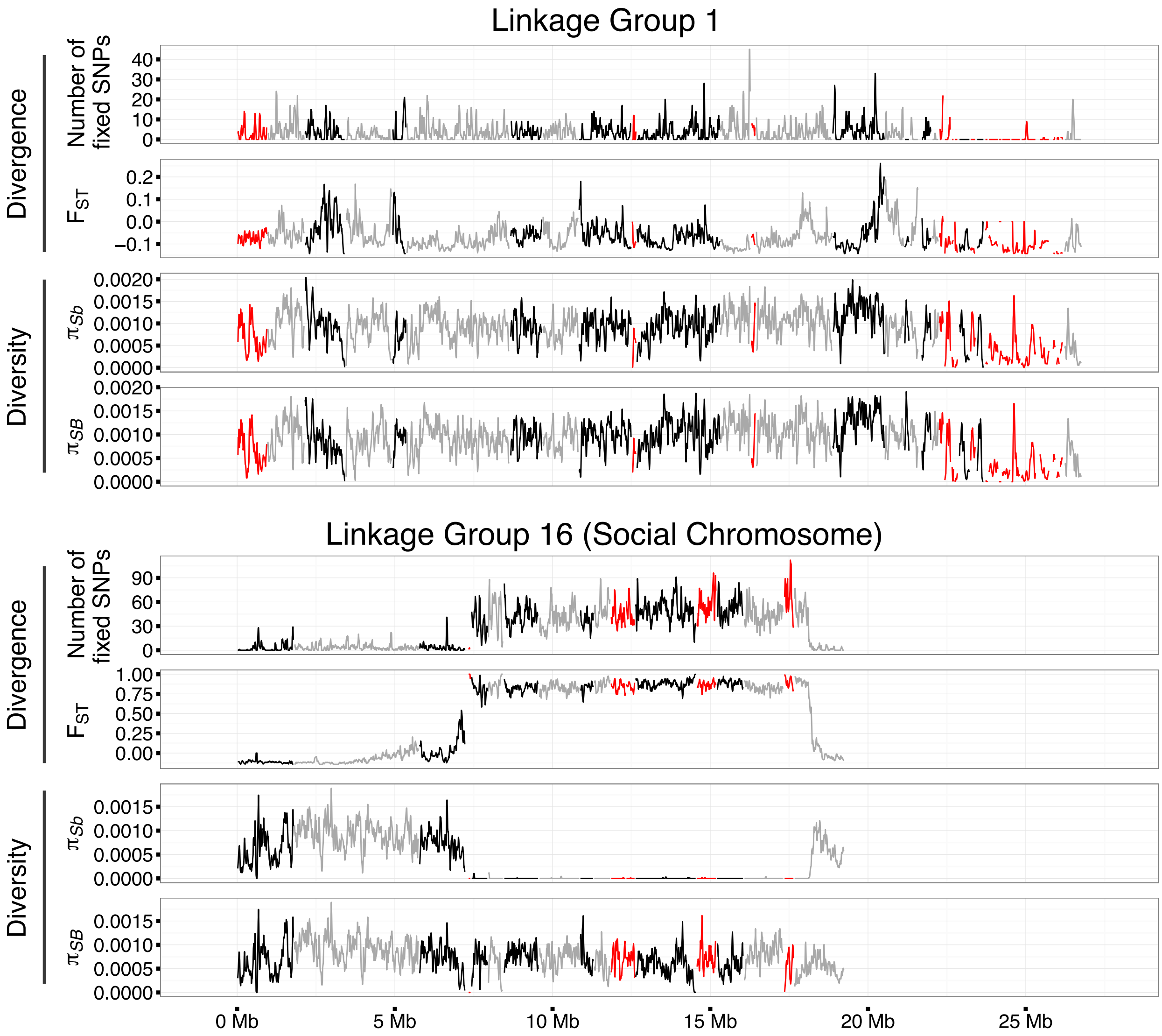

Figure S2: Differentiation and diversity in linkage group 1 and linkage group 16 (the social chromosome) in 30 kb sliding windows with a step of 10 kb. Measurements include the number of fixed SNPs per window, F_{ST}, nucleotide diversity among SB individuals (π_{SB}) and nucleotide diversity among Sb individuals (π_{Sb}). Different colors represent different scaffolds. Scaffolds in red have unknown orientation.

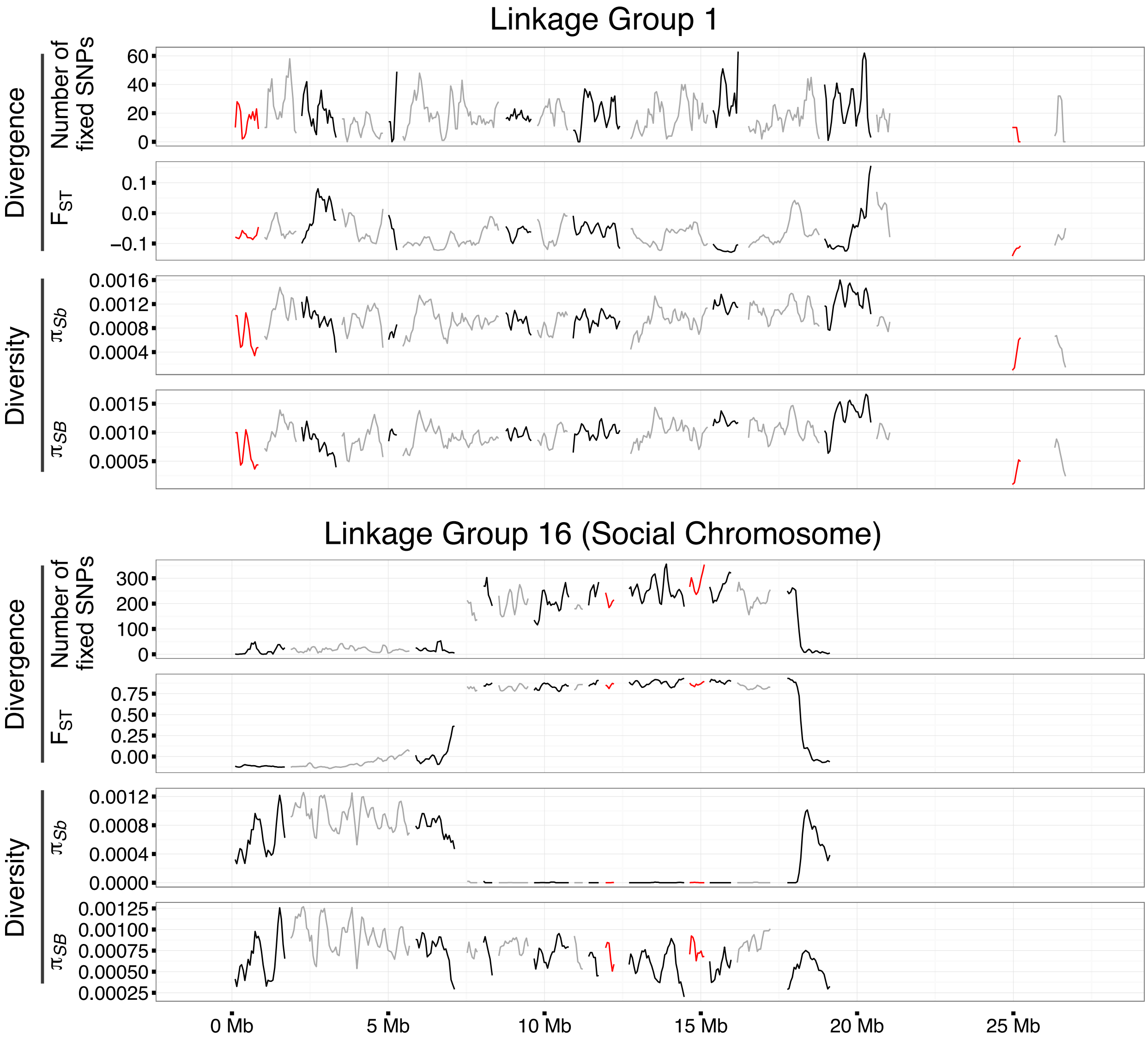

Figure S3: Differentiation and diversity in linkage group 1 and linkage group 16 (the social chromosome) in 15 kb sliding windows with a step of 50 kb. Measurements include the number of fixed SNPs per window, F_{ST}, nucleotide diversity among SB individuals (π_{SB}) and nucleotide diversity among Sb individuals (π_{Sb}). Different colors represent different scaffolds. Scaffolds in red have unknown orientation.

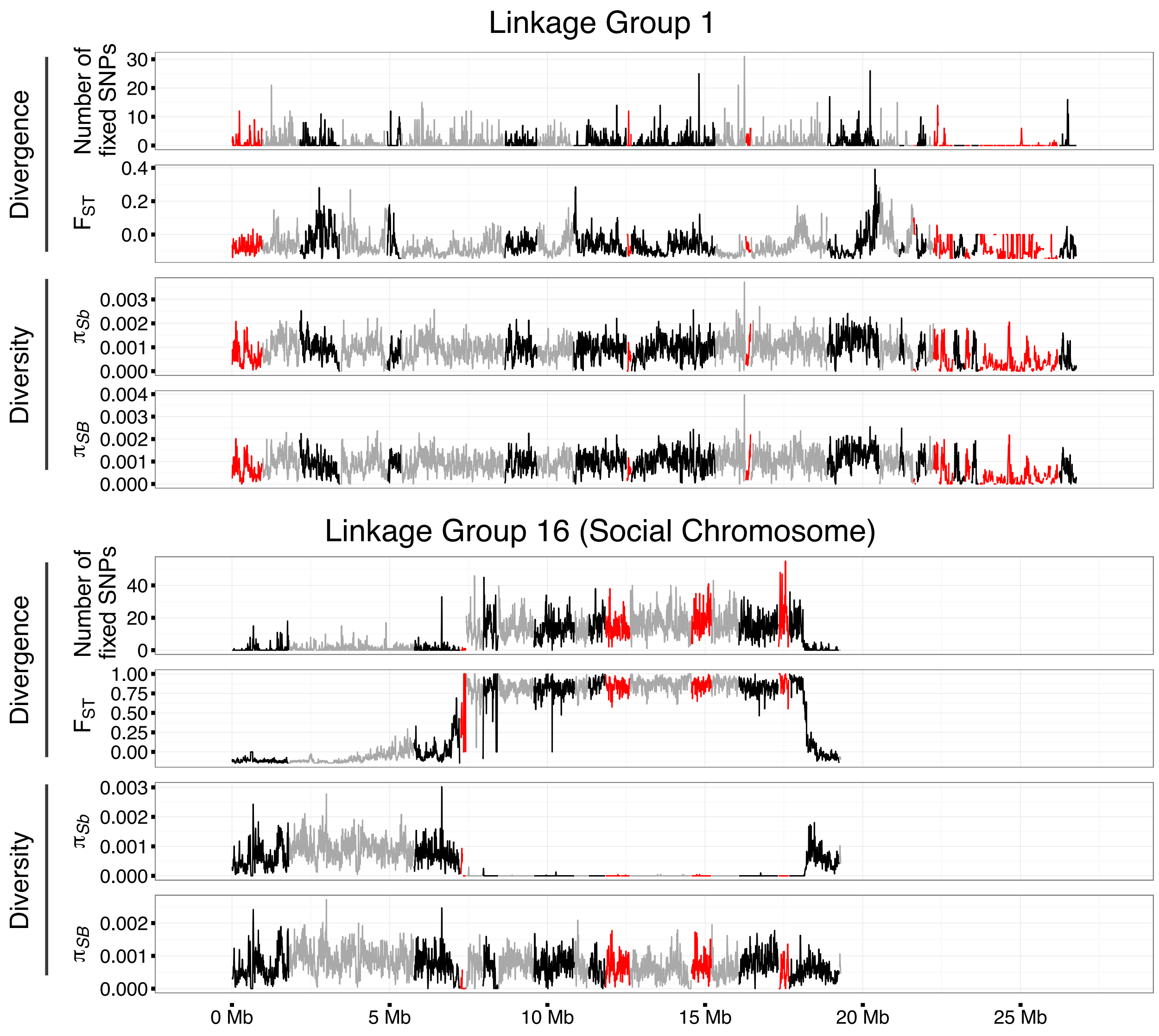

Figure S4: Differentiation and diversity in linkage group 1 and linkage group 16 (the social chromosome) in 10 kb sliding windows with a step of 5 kb. Measurements include the number of SNPs with a fixed difference per window, F_{ST}, nucleotide diversity among SB individuals (π_{SB}) and nucleotide diversity among Sb individuals (π_{Sb}). Different colors represent different scaffolds. Scaffolds in red have unknown orientation.

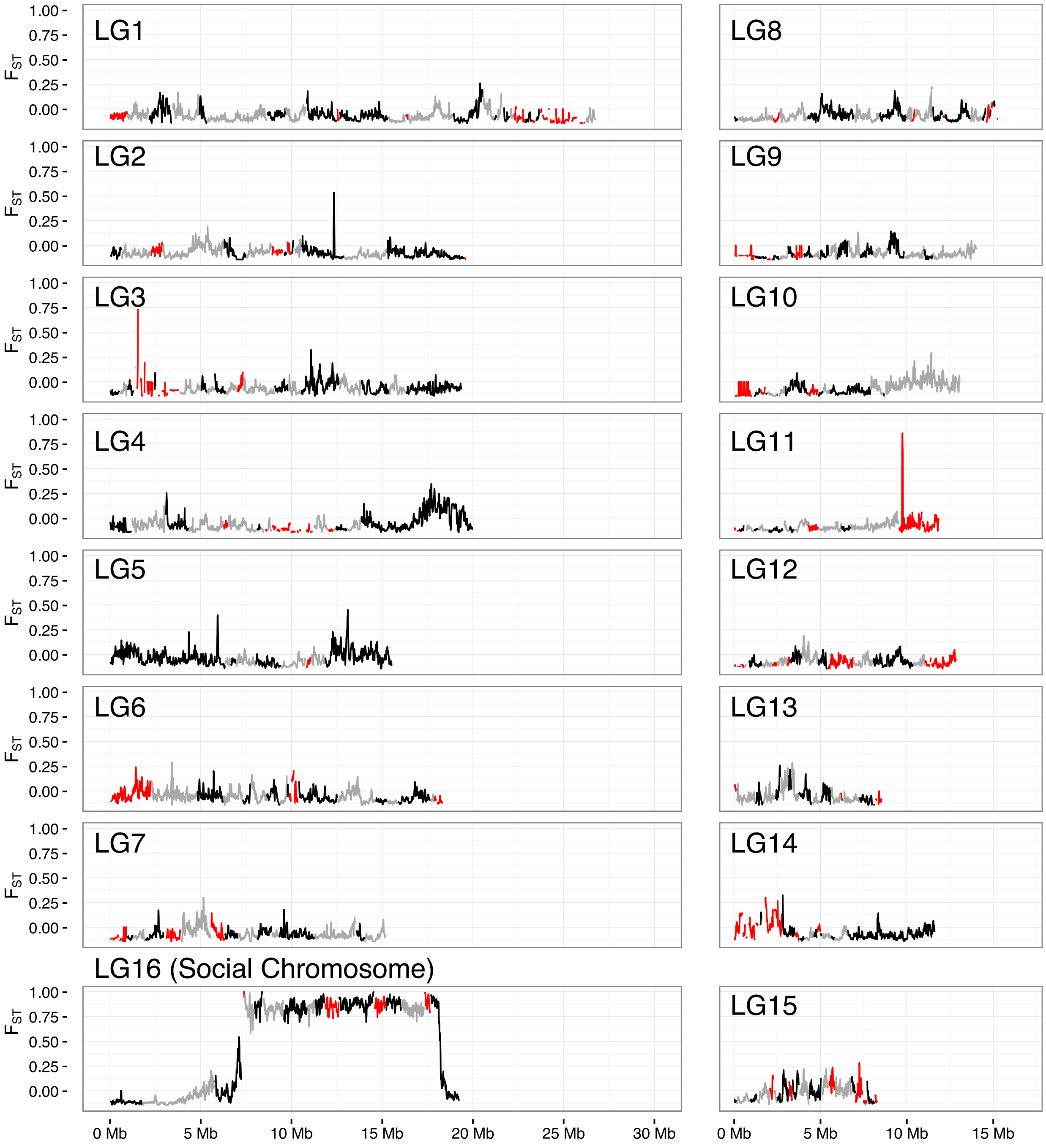

Figure S5: F_{ST} in 30kb sliding windows with a step of 10kb along scaffolds mapped to all linkage groups (LG1 to LG16). Different colors represent different scaffolds. Scaffolds in red have unknown orientation.

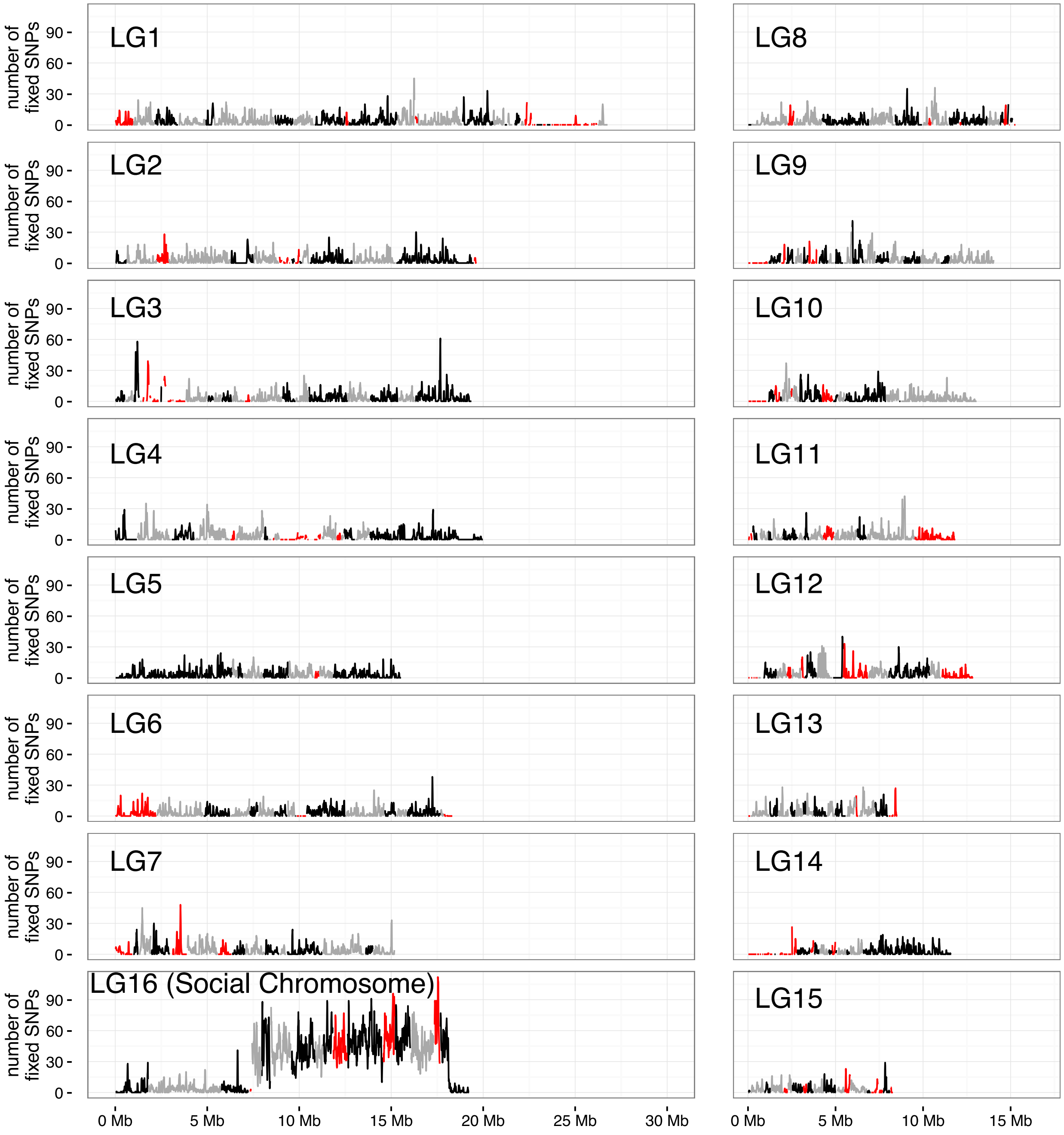

Figure S6: Number of SNPs with a fixed difference per 30kb sliding windows with a step of 10kb along scaffolds mapped to all linkage groups (LG1 to LG16). Different colors represent different scaffolds. Scaffolds in red have unknown orientation.

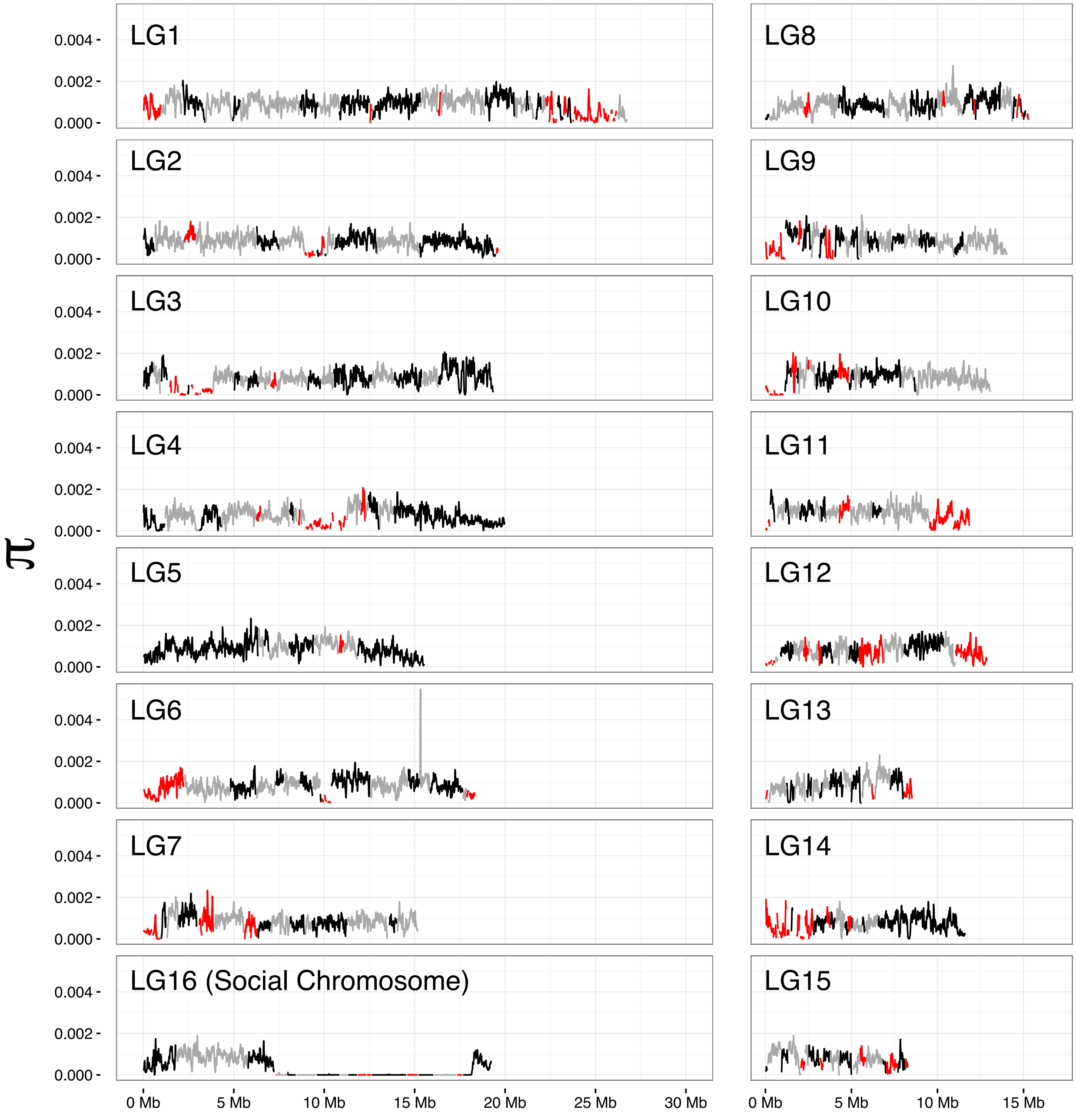

Figure S7: Nucleotide diversity among *Sb* individuals (π) in 30kb sliding windows with a step of 10kb along scaffolds mapped to all linkage groups (LG1 to LG16). Different colors represent different scaffolds. Scaffolds in red have unknown orientation.

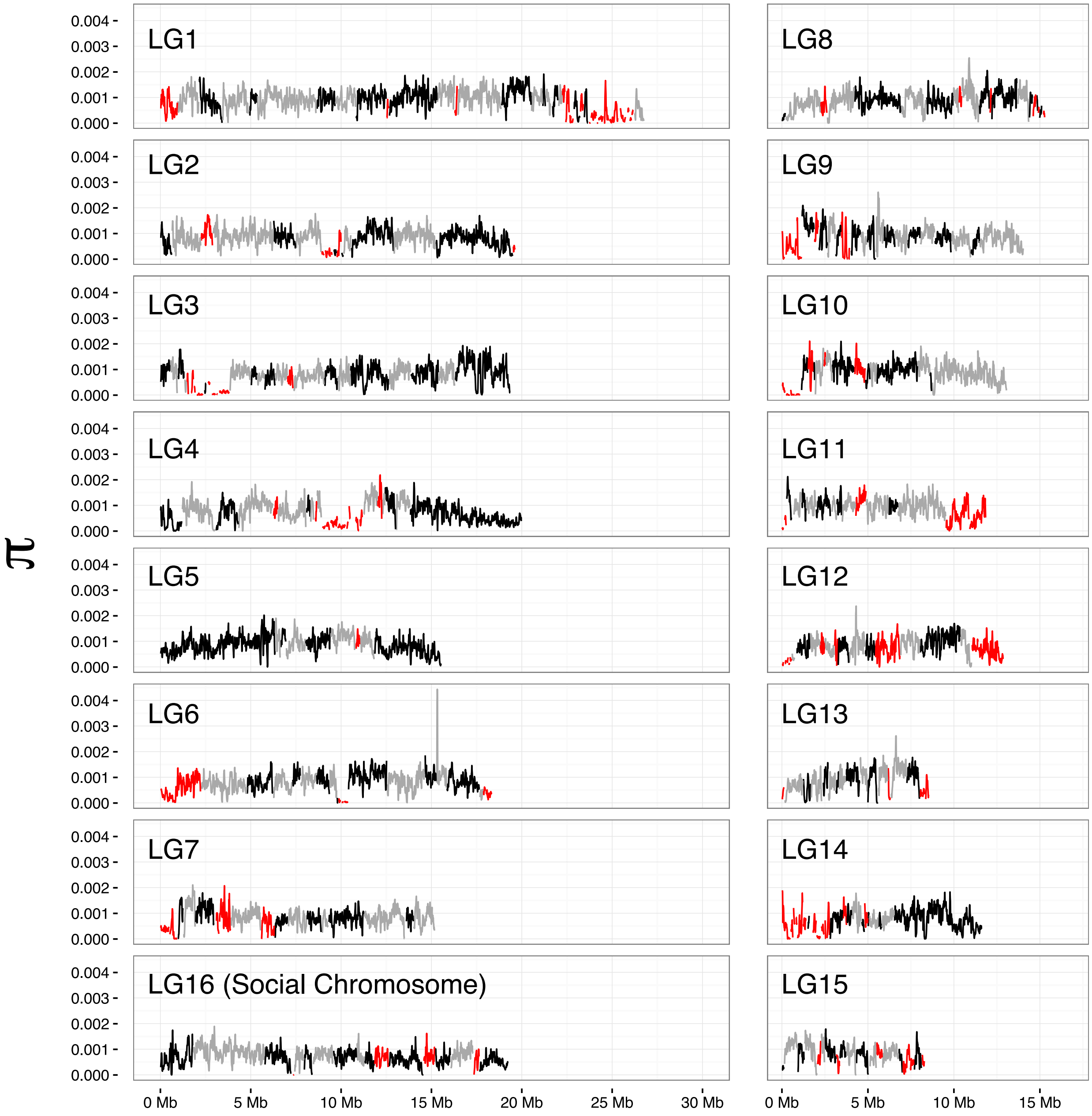

Figure S8: Nucleotide diversity among *SB* individuals (π) in 30kb sliding windows with a step of 10kb along scaffolds mapped to all linkage groups (LG1 to LG16). Different colors represent different scaffolds. Scaffolds in red have unknown orientation.

(A) SNP sites

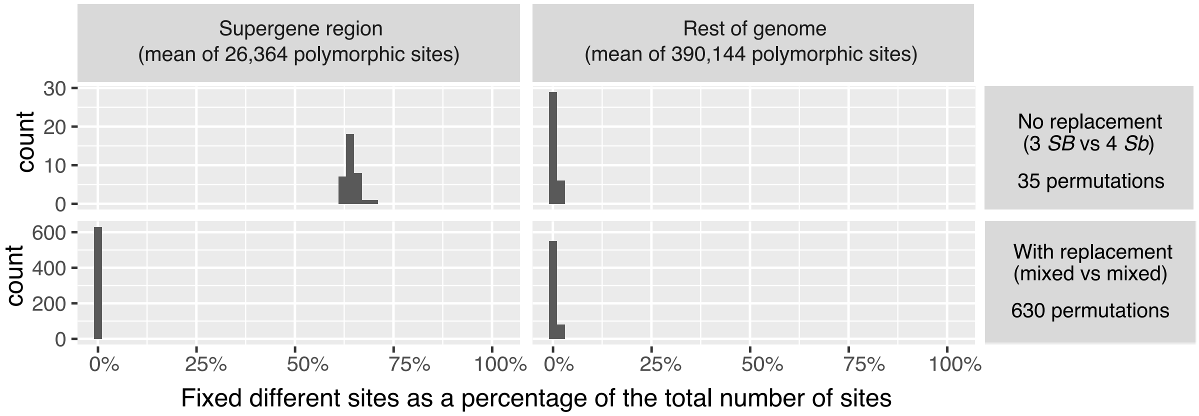

(B) Short Tandem Repeats sites

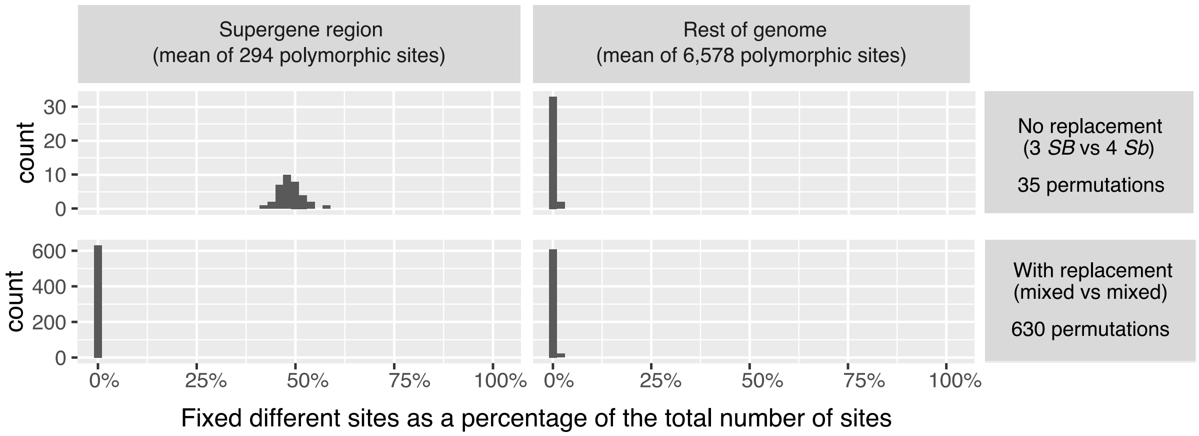

Figure S9: We used permutation tests to ensure that the fixed differences between the group of *SB* individuals and the group of *Sb* individuals were not the result of an arbitrary grouping of individuals. First, we counted the number of fixed differences between groups of *SB* and groups of *Sb* individuals (“no replacement” strategy). Then, we counted the number of fixed differences between groups including individuals of both genotypes (“with replacement” strategy). The figure shows that, in the supergene region, we observe a high number of fixed differences only when the individuals are grouped by genotype. For all permutations, we only used the 7 low coverage individuals; we only included comparisons between non-sibling individuals, which limited in size of the groups. In the “no replacement” treatment, we compared all groups with 3 *SB* individuals with all groups with 4 *Sb* individuals. In the “with replacement” treatment, we compared groups with 2 *SB* and 2 *Sb* individuals with groups with 2 *Sb* and 1 *SB* individuals.

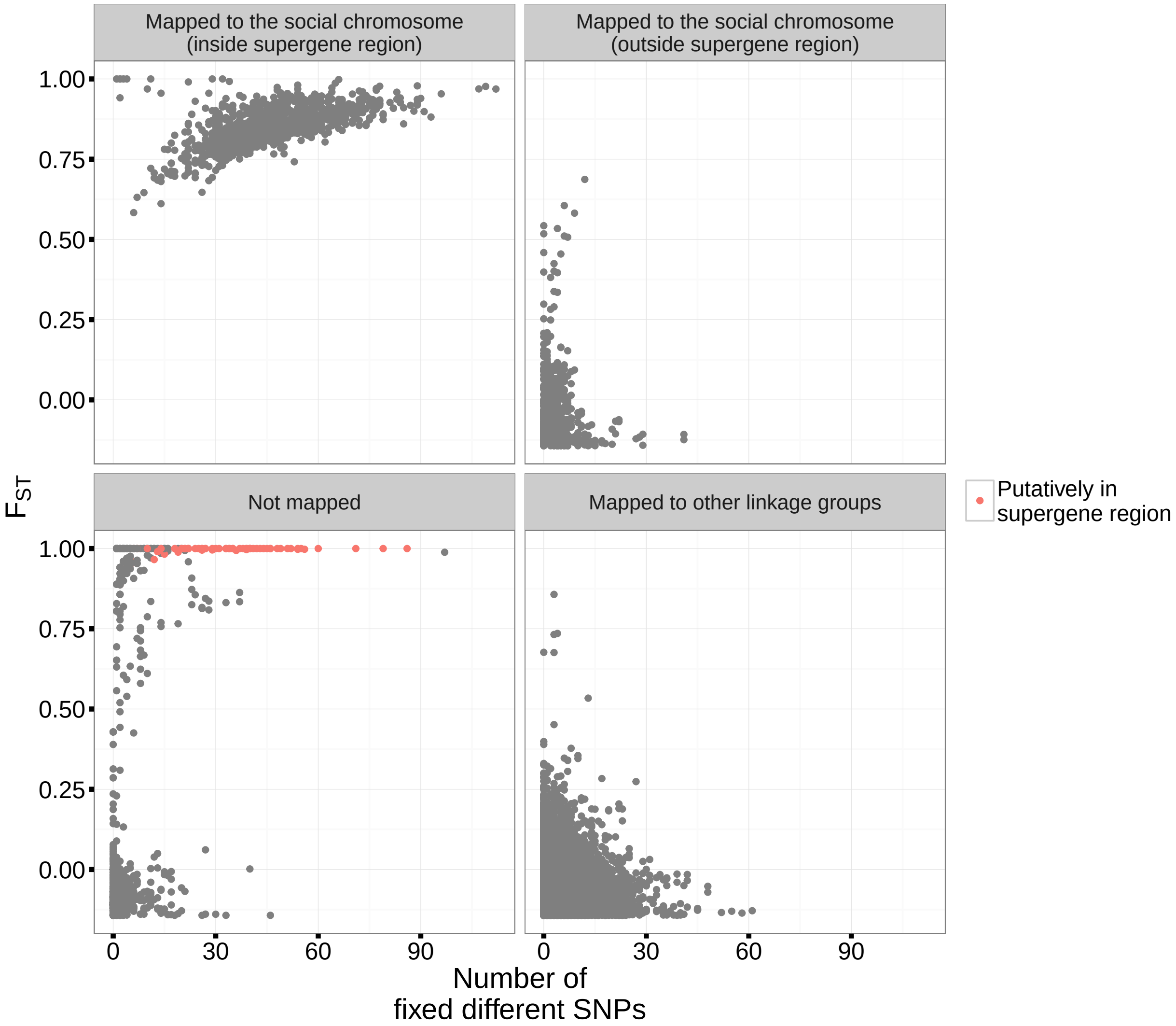

Figure S10: Signature of genetic differentiation between *SB* and *Sb* individuals in different regions of the genome. Each point shows the number of SNP sites with different alleles fixed in each genotype group and the fixation index, F_{ST}, in a 30kb window (sliding along each scaffold with a step of 10kb). The colored points are windows in the 5 large non-mapped scaffolds (length $> 100kb$) with at least one window of F_{ST} larger than 0.75 and more than 25 SNP fixed different SNP sites.

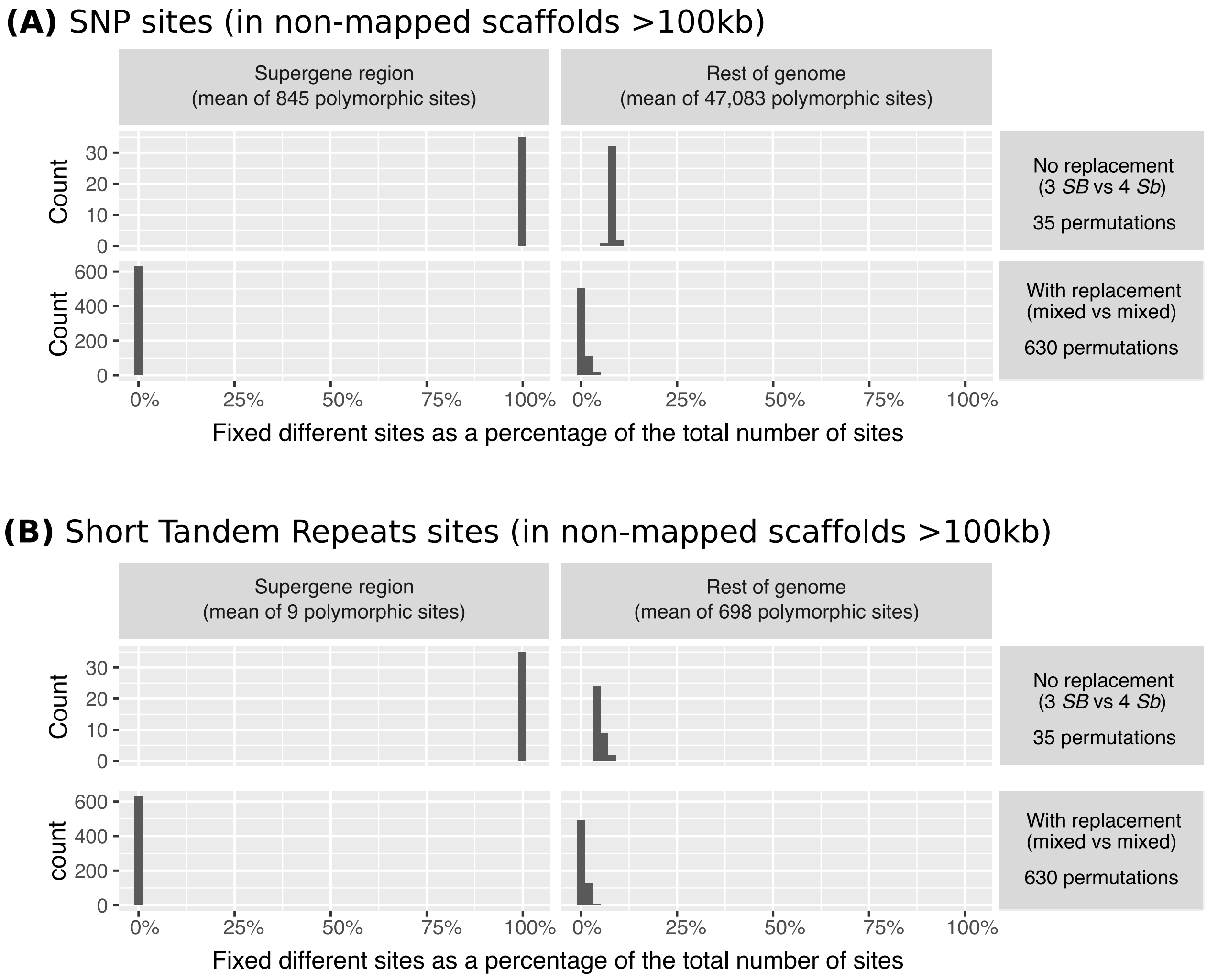

Figure S11: Permutation tests showing that, in the scaffolds that putatively belong to the supergene region, we observe a high number of fixed differences only when we group the individuals by genotype. The permutation strategy used in this analysis is explained in Fig. S9, Supporting Information.

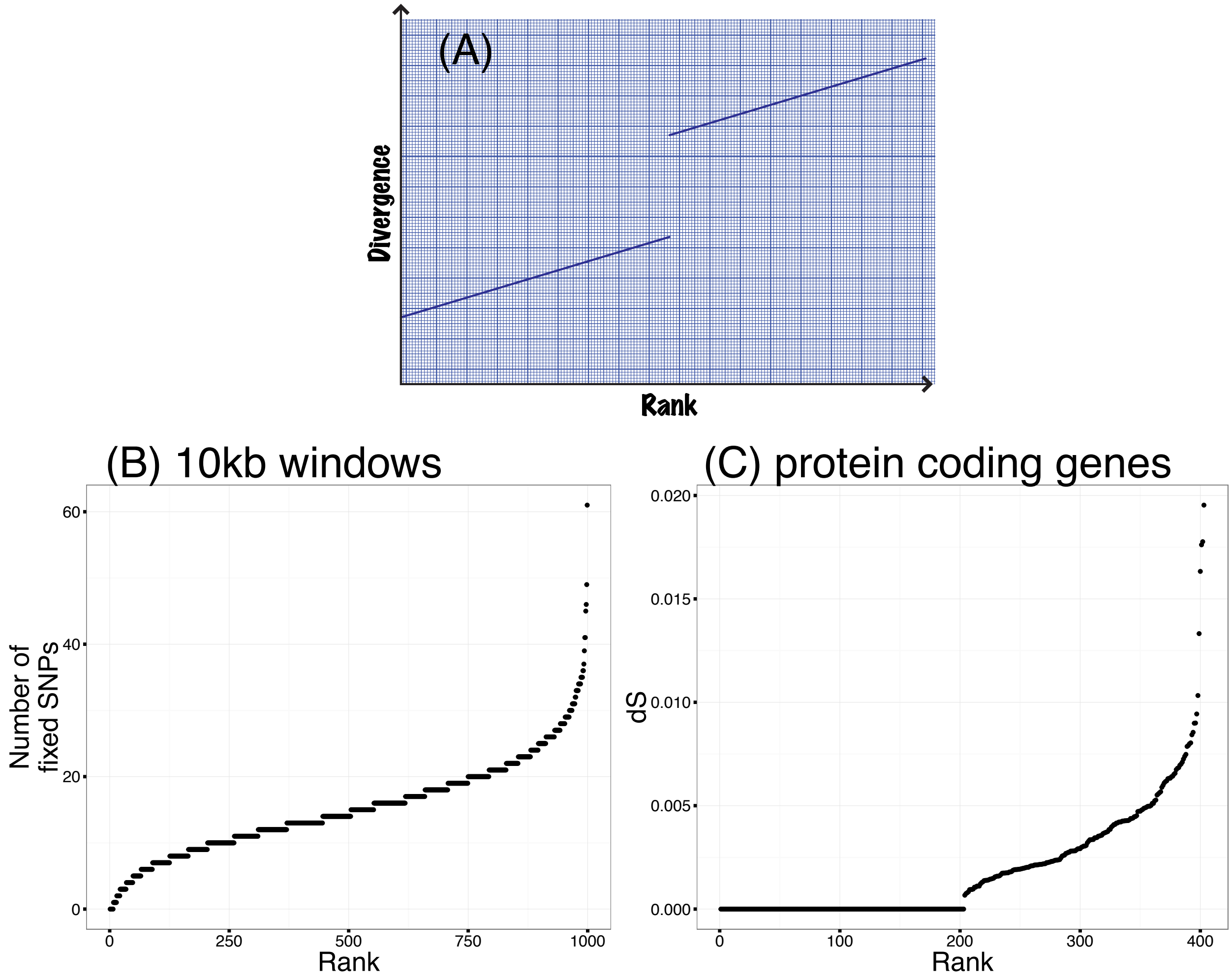

Figure S12: Absence of strata with non-overlapping ranges of divergence between SB and Sb. (A) Illustrative example of the ranked distribution of divergence in a system with two strata. (B) Ranked distribution of SNPs with fixed differences between *SB* and *Sb* individuals in 10kb non-overlapping sliding windows. (C) Ranked distribution of the rate of nonsynonymous substitutions between SB and Sb, *dS*. The distributions of the measurements of divergence in (B) and (C) are continuous, rather than grouping into non-overlapping clusters.

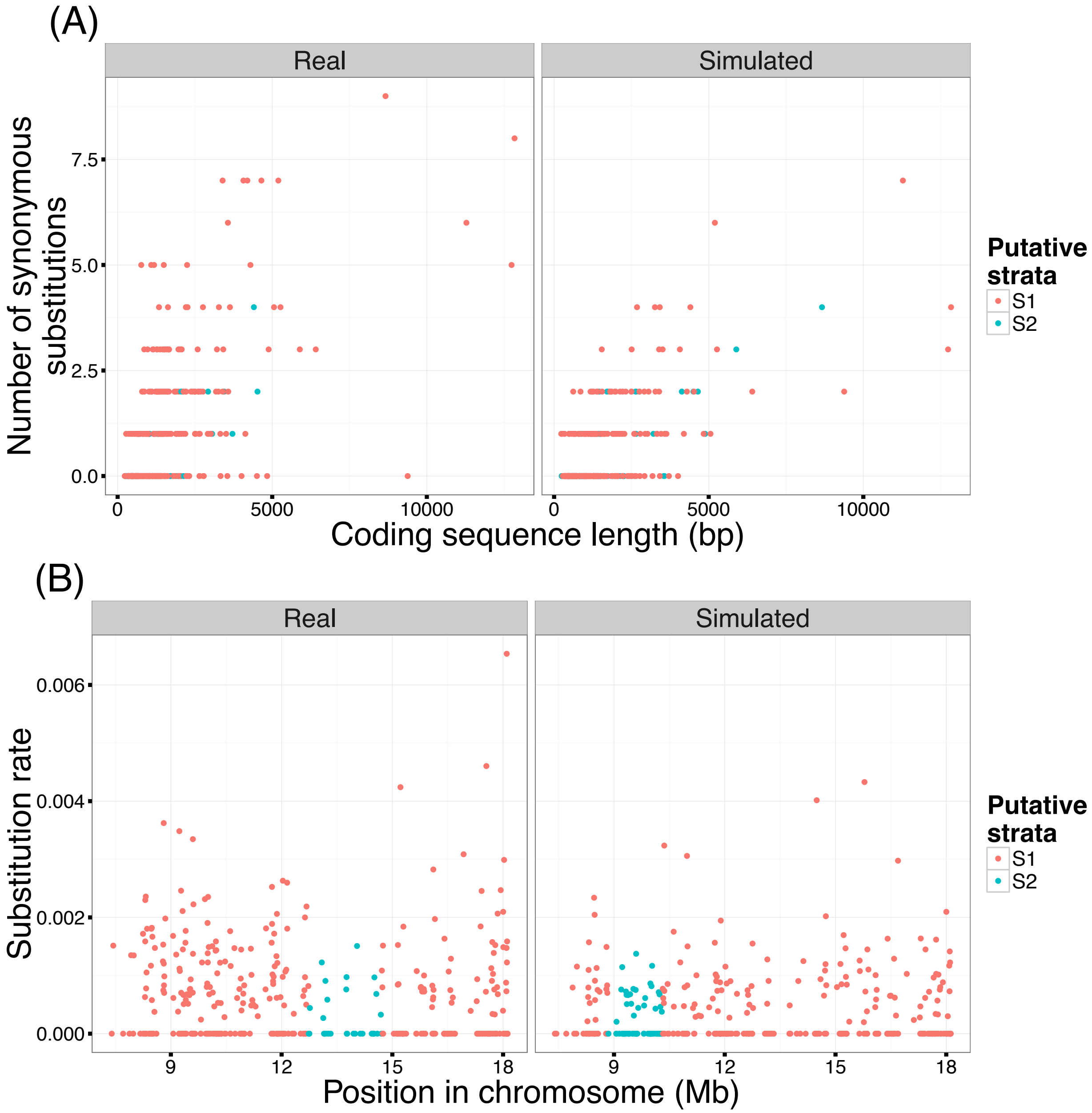

Figure S13: Differences in the rate of synonymous substitutions (dS) between putative evolutionary strata in the real data and in an example of a simulation. (A) Real and simulated number of synonymous mutations in protein-coding sequences relative to their sizes. (B) Real and simulated distribution of the rate of synonymous mutations along the chromosome. Putative strata were defined as the group of neighbouring genes with the most significant differences in dS from the rest of the genes, using Welch t-tests.

Table S1: Number of variant sites detected with Cortex (Iqbal *et al.* 2012) in each group of samples; number of variants with different alleles fixed in each of the *SB* and *Sb* groups. Indels were classified as insertions or deletions relative to the reference assembly.

		Number of Variant Sites in Each Group			Variants Fixed Between *SB* and *Sb*	
Region	Variant Type	All Individuals	*SB*	*Sb*	Number	% of Total
Mapped Variants	SNP	476 758	458 047	433 903	15 404	80%
	Indel	86 692	87 221	82 891	2 336	78%
	Inversion	21	23	21	0	
Social Chromosome (LG16)	SNP	45 363	31 273	15 211	15 378	80%
	Indel	7 622	5 816	2 970	2 331	78%
	Inversion	0	1	0	0	
Supergene Region	SNP	29 857	16 033	41	15 367	79%
	Indel	4 737	2 841	14	2 329	77%
	Inversion	0	1	0	0	
Non-mapped	SNP	54 670	51 026	50 236	3933	20%
	Indel	10 332	10 168	10 003	670	22%
	Inversion	3	2	3	0	
Whole Genome	SNP	531 428	509 073	484 139	19 337	100%
	Indel	97 024	97 389	92 894	3 006	100%
	Inversion	24	25	24	0	

Table S2: Number of polymorphic and non-polymorphic short tandem repeat (STR) sites detected with lobSTR (Gymrek *et al.* 2012) in each group of samples; number of variants with different alleles fixed in each of the *SB* and *Sb* groups. The number of sites in each group is larger than among all individuals because our filter removed any site where one or more individual had an uncertain call (the more individuals are included in the group, the more likely one of the individuals has an uncertain call).

		Number of Variant Sites in Each Group			Variants Fixed Between *SB* and *Sb*	
Region	Site Type	All Individuals	*SB*	*Sb*	Number	% of Total
Mapped variants	Polymorphic	7 589	13 487	12 118	111	75%
	Non-polymorphic	21 420	34 410	33 768		
Social Chromosome (LG16)	Polymorphic	585	1 019	456	111	75%
	Non-polymorphic	1 402	2 729	2 859		
Supergene Region	Polymorphic	324	537	0	111	75%
	Non-polymorphic	709	1 557	1 779		
Non-mapped	Polymorphic	796	1 292	1 238	37	25%
	Non-polymorphic	6 876	10 733	10 361		
Whole Genome	Polymorphic	8 385	14 779	13 356	148	100%
	Non-polymorphic	28 296	45 143	44 129		

Table S3: Visual inspection of SNP and indel positions variable among *Sb* individuals in the supergene region. Using IGV, we inspected whole genome alignments of each individual at those positions. We accepted the genotypes called by Cortex if they were unambiguously supported by the alignment (where filter is PASS). The asterisk (*) indicates positions where the genotype calling seems to have been disrupted by a structural variant (where reads mapping close to the variant position have pairs mapping elsewhere in the genome, indicative of an insertion).

Index	Scaffold	Position	Reference	Alternative	Filter
	NW_011803911.1	203 526	G	A	Incorrect allele call in one or more individuals
	NW_011803911.1	378 670	AT	A	Incorrect allele call in one or more individuals
1	NW_011803911.1	569 505	A	G	PASS
2	NW_011803911.1	569 697	A	G	PASS
3	NW_011803911.1	569 827	A	G	PASS
4	NW_011803911.1	569 978	T	C	PASS
5	NW_011803911.1	570 689	A	G	PASS
6	NW_011803911.1	571 294	ATCTAT	A	PASS
7	NW_011803911.1	571 395	C	T	PASS
8	NW_011803911.1	571 519	AATA	A	PASS
9	NW_011803911.1	571 541	C	T	PASS
	NW_011800721.1	410 652	C	CT	Incorrect allele call in one or more individuals
10	NW_011800721.1	468 563	C	T	PASS
11	NW_011800721.1	469 032	C	T	PASS
12	NW_011800721.1	472 291	T	A	PASS
13	NW_011800721.1	472 952	A	G	PASS
14	NW_011800721.1	473 520	A	T	PASS
15	NW_011800721.1	477 385	TTG	T	PASS
16	NW_011800721.1	478 358	G	A	PASS
17	NW_011800721.1	478 448	G	A	PASS
18	NW_011800721.1	48 329	G	A	PASS
19	NW_011800721.1	490 241	GA	G	PASS
20	NW_011800721.1	494 458	A	AAATG	PASS
21	NW_011800721.1	497 480	C	G	PASS
	NW_011799419.1	328 351	A	ATCA	Incorrect allele call in one or more individuals*
	NW_011799419.1	380 782	A	G	Incorrect allele call in one or more individuals
22	NW_011799419.1	433 685	C	T	PASS
	NW_011799419.1	591 946	C	T	Incorrect allele call in one or more individuals
	NW_011799419.1	806 516	CAAC	C	Incorrect allele call in one or more individuals
23	NW_011801243.1	66 953	G	GA	PASS
24	NW_011801243.1	397 534	T	G	PASS
25	NW_011801243.1	540 144	T	TA	PASS
26	NW_011801243.1	705 693	G	A	PASS
27	NW_011801243.1	706 439	C	A	PASS
28	NW_011801243.1	706 697	T	C	PASS
29	NW_011795711.1	405 472	C	T	PASS
30	NW_011797558.1	203 161	C	G	PASS
	NW_011794567.1	169 810	TT	T	Incorrect allele call in one or more individuals
	NW_011794567.1	169 816	C	T	Incorrect allele call in one or more individuals
	NW_011794567.1	345 652	A	ACT	Incorrect allele call in one or more individuals
31	NW_011794567.1	878 315	G	A	PASS
32	NW_011794567.1	971 265	A	G	PASS
33	NW_011794567.1	981 981	G	A	PASS
34	NW_011794567.1	1 672 010	G	A	PASS
35	NW_011794567.1	1 726 264	A	G	PASS
	NW_011794567.1	1 878 701	A	T	Incorrect allele call in one or more individuals
	NW_011795727.1	52 980	AA	A	Incorrect allele call in one or more individuals
36	NW_011795727.1	175 318	C	T	PASS
37	NW_011795727.1	263 996	C	T	PASS
38	NW_011794844.1	554 175	G	T	PASS
39	NW_011794844.1	554 371	C	T	PASS
40	NW_011794844.1	554 533	G	T	PASS
41	NW_011794844.1	554 590	A	G	PASS
	NW_011794844.1	554 988	T	C	Incorrect allele call in one or more individuals*
